# Unraveling the Phase Behavior, Mechanical Stability, and Protein Reconstitution Properties of Polymer–Lipid Hybrid Vesicles

**DOI:** 10.1021/acs.biomac.3c00498

**Published:** 2023-08-04

**Authors:** Wagner A. Müller, Paul A. Beales, André R. Muniz, Lars J. C. Jeuken

**Affiliations:** † Department of Chemical Engineering, Universidade Federal do Rio Grande do Sul, Porto Alegre 90035-003, Brazil; ‡ School of Chemistry and Astbury Centre for Structural Molecular Biology, University of Leeds, Leeds LS2 9JT, U.K.; § Leiden Institute of Chemistry, University Leiden, PO Box 9502, 2300RA Leiden, The Netherlands

## Abstract

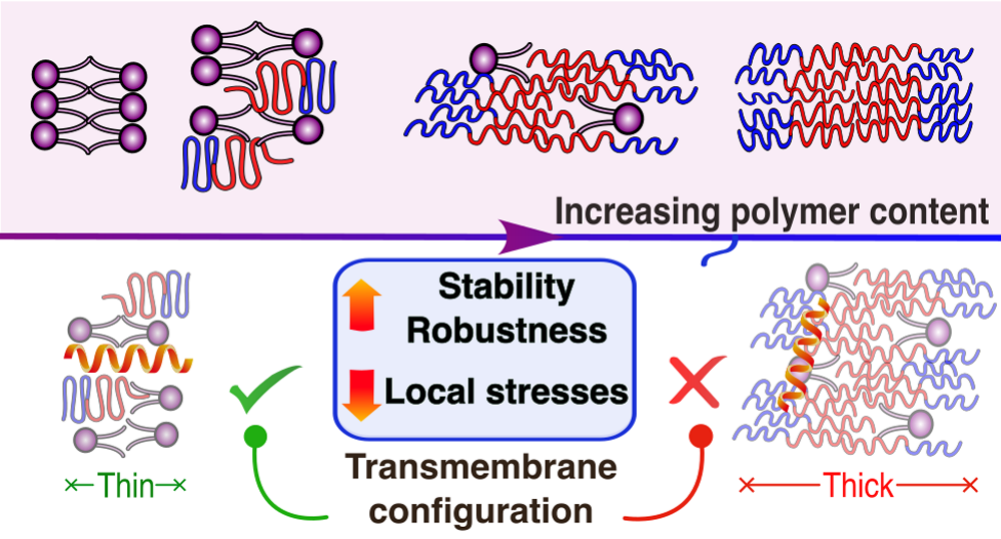

Hybrid vesicles consisting of natural phospholipids and synthetic amphiphilic copolymers have shown remarkable material properties and potential for biotechnology, combining the robustness of polymers with the biocompatibility of phospholipid membranes. To predict and optimize the mixing behavior of lipids and copolymers, as well as understand the interaction between the hybrid membrane and macromolecules like membrane proteins, a comprehensive understanding at the molecular level is essential. This can be achieved by a combination of molecular dynamics simulations and experiments. Here, simulations of POPC and PBD_22_-*b*-PEO_14_ hybrid membranes are shown, uncovering different copolymer configurations depending on the polymer-to-lipid ratio. High polymer concentrations created thicker membranes with an extended polymer conformation, while high lipid content led to the collapse of the polymer chain. High concentrations of polymer were further correlated with a decreased area compression modulus and altered lateral pressure profiles, hypothesized to result in the experimentally observed improvement in membrane protein reconstitution and resistance toward destabilization by detergents. Finally, simulations of a WALP peptide embedded in the bilayer showed that only membranes with up to 50% polymer content favored a transmembrane configuration. These simulations correlate with previous and new experimental results and provide a deeper understanding of the properties of lipid-copolymer hybrid membranes.

## Introduction

Phospholipid vesicles, or liposomes, have many biotechnological applications, including in the delivery of drugs and vaccines,^1,2^ diagnostic imaging,^3^ biosensors,^4^ and nanoreactors.^5^ A key drawback of these vesicles is their limited stability, which is caused by various factors, including the formation of transient pores in the lipid bilayer and the oxidation of the acyl chains.^6^ To address this, polymersomes (formed by the spontaneous self-assembly of amphiphilic copolymers) have been proposed as an alternative in recent decades.^7,8^ Polymersomes are usually more resistant and exhibit a wider range of physicochemical properties due to the diversity of polymers that can be used to form them. However, besides some notable exceptions,^9−11^ polymer bilayers are not suitable for the functional incorporation of membrane proteins,^12,13^ which evolved to be biocompatible with phospholipids and not with copolymers. Hence, genetic modifications of the proteins or changes in the membrane structure are necessary to make polymersomes a more viable option for biotechnological applications.

In the past decade, hybrid vesicles (HVs) composed of mixtures of phospholipids and copolymers have been studied, aiming to combine the best properties of both components: the biocompatibility of liposomes and the robustness of polymersomes.^14−16^ HVs composed of 1-palmitoyl-2-oleoyl-*sn*-glycero-3-phosphocholine (POPC) and poly(1,2-butadiene)-*b*-poly(ethylene oxide) (PBD_22_-*b*-PEO_14_) were found to efficiently incorporate cytochrome *bo*_3_, with a 50/50 mol % ratio providing the best cost-benefit scenario considering the balance between long-term stability and initial activity of the protein.^13,17^ Also, when reconstituting cytochrome *bo*_3_ from styrene-maleic-acid lipid particles, no surfactants were required for HVs, whereas pure liposomes necessitated the addition of a detergent to effectively incorporate this protein.^18^ The addition of PBD_22_-*b*-PEO_14_ in a DOPC vesicle has been shown to also improve the efficiency of protein folding and reconstitution into the membrane.^19^ Enhanced protein activity in PDMS-*g*-PEO and soy-PC HVs was also reported.^20^ Therefore, despite the distinct structural characteristics of lipids and copolymers, HVs have demonstrated various examples of equivalent/superior performance when compared to pure liposomes and polymersomes, showcasing their unique advantages. These observations have led to an increased interest in the mixing behavior of lipids and polymers,^2,21,22^ and a need to optimize the vesicle environment through a better understanding of the relationship between a hybrid membrane’s chemical composition and its material properties.

Experimental techniques have been widely used to explore the physicochemical properties of pure and hybrid membranes, but some phenomena are not easily understood through experiments alone. Molecular dynamics (MD) simulations allow for the analysis of atomistic trajectories, providing valuable insights that can complement experimental data and offer a deeper understanding of relevant phenomena at the molecular level.^23^ Although the use of MD applied to HVs holds great potential, the use of computational techniques in the study of these membranes is still in its early stages of development. Recently, all-atom (AA) MD simulations were performed to investigate HVs composed of DOPC and PBD-*b*-PEO, with polymer fractions of 10 and 20 mol %.^24^ These simulations offered valuable insights into the molecular structure of these vesicles. However, atomistic simulations face limitations in their ability to analyze systems with more than hundreds of thousands of atoms (a common occurrence in biochemistry^25^) over long time scales. To overcome this limitation, coarse-graining (CG) techniques can be utilized to access longer time and length scales by simplifying some of the atomistic degrees of freedom. To our knowledge, this technique has not been employed in the context of hybrid membranes until now.

The objective of this study is to obtain a deeper understanding of the structure and properties of HVs, in particular POPC and PBD_22_-*b*-PEO_14_ HVs, by combining experiments and mesoscale MD simulations. Through a comprehensive and integrated analysis of various physicochemical, mechanical, and biochemical metrics, we strive to gain valuable insights into the membrane properties, and their correlation with previous experimental findings. The POPC/PBD_22_-*b*-PEO_14_ system was chosen due to its extensive previous characterization using various experimental techniques, such as small-angle X-ray scattering (SAXS) and cryo-electron tomography,^22^ general polarization measurements of Laurdan,^26^ confocal fluorescence microscopy^27^ and flow cytometry,^28^ which raised several questions regarding the observed phenomena that can benefit from MD simulations. A coarse-graining method using the Martini force field (FF) was employed following its widespread use for biomolecules. As Martini currently lacks a parameterization for the analyzed copolymer, a model for this molecule was first developed. Qualitative and quantitative metrics were evaluated to study the fundamental interactions of lipids and copolymers within the HVs. Furthermore, the incorporation of a WALP peptide in HVs was assessed to investigate peptide/membrane interactions. Fluorescence experiments were performed to evaluate the leakage of HVs in the presence of detergent, aiming to determine if increasing the concentration of polymers enhances the stability of the membrane when surfactants are added. The comparison between simulation and experimental results allowed the examination of diverse phenomena in HVs, such as the presence of different spatial configurations in homogeneous membranes^22^ and improved incorporation of membrane proteins into HVs.^18,19^

## Computational Methods

### Molecular Dynamics Simulations

Because of the characteristic length scale of biological membranes, a CG approach was adopted to study the HVs. The Martini 3 force field^29^ was applied due to its success in reproducing key aspects of POPC membranes.^30,31^ Additionally, the Martini 3 version has demonstrated notable advancements in accurately describing the behavior of peptides and transmembrane proteins within bilayers.^29^ A cutoff of 1.2 nm was employed for both electrostatic and nonbonded interactions. All simulations were performed using the open-source Gromacs package,^32^ with periodic boundary conditions in all dimensions.

### Parametrization of PBD-*b*-PEO

Currently, there is no Martini parametrization for the PBD-*b*-PEO copolymer. Therefore, we developed a new CG model for this system, following protocols previously defined in the literature and adhering to Martini’s best practices for similar molecules.^29,33^ The PEO chain was modeled using SN3a beads, which are appropriate for ether molecules, while the PBD monomer was modeled with TC2r and TC4r beads for single and double-bonded carbon atoms, respectively. Bonded parameters were obtained from AA simulations using the OPLS-AA FF.^34^ The model was able to predict qualitative features of hybrid membranes, including the homogeneous mixing of polymers and phospholipids^26^ and the absence of polar particles in the hydrophobic core.^33,35^ The full details of the parametrization procedure can be found in Section S1 of the Supporting Information. The broad applicability of the Martini force field makes the proposed model transferable and suitable for investigating the interactions of this copolymer with various molecules.

### Initial System Setup and Analysis

**Figure 1:**
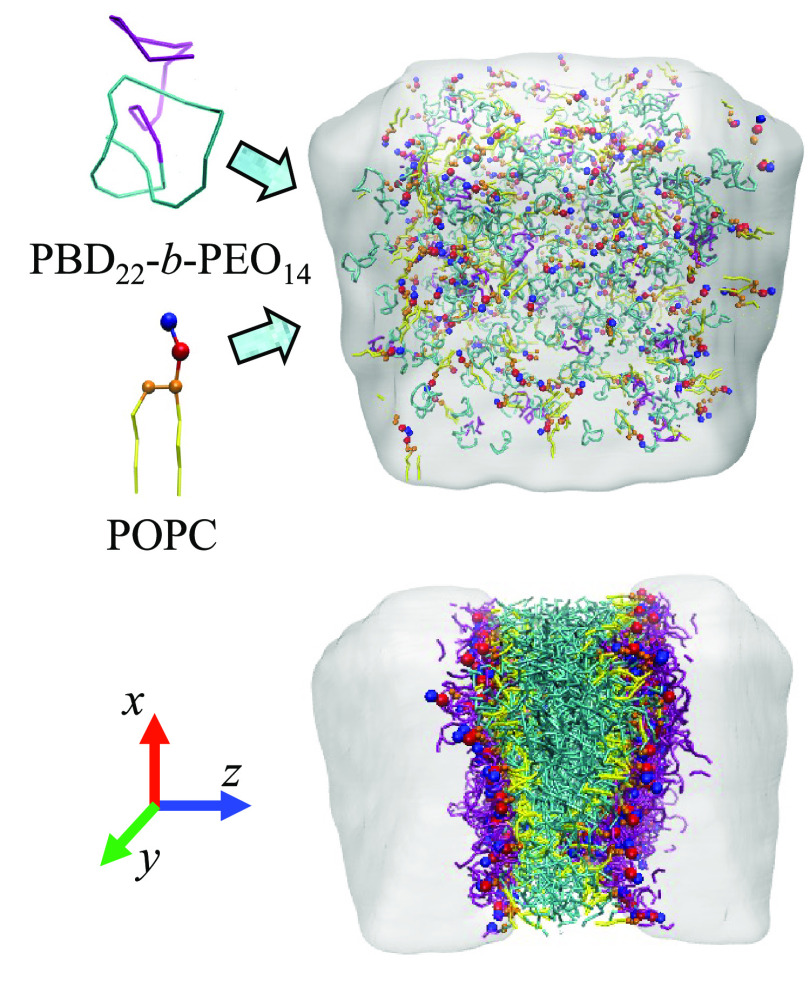
System representation and workflow of the simulations: POPC and PBD_22_-*b*-PEO_14_ were randomly distributed in a solvent medium and spontaneously assembled into organized bilayers/vesicles.

Different systems were built by randomly placing Martini water beads and POPC and/or PBD_22_-*b*-PEO_14_ in a simulation box, as shown in Figure 1. To analyze bilayers with different polymer/lipid ratios, the concentration of polymer was systematically altered in increments of 25% from 0 to 100% (mol %). Also, to verify that the developed parameterization is capable of spontaneously forming both bilayers and vesicles, systems with varying degrees of hydration were created. To form bilayers, 500 molecules were mixed with 10^4^ water beads, while for vesicles, 2000 molecules were solvated with 10^5^ water beads. Although the degree of hydration varied between the two systems, both conditions were fully hydrated (≥40 real water molecules per lipid/copolymer^36^). These systems were energy-minimized using the steepest descent algorithm, followed by a 200 ns equilibration at 4 independent temperatures (290 to 350 K with increments of 20 K) in the isobaric isothermal ensemble (*NPT*, *P* = 1 atm) with isotropic pressure coupling. A time step of 10 fs was applied during equilibration to avoid instabilities during the initial stages of the simulations. Spontaneously self-assembled bilayers/vesicles (as illustrated in Figure 1) were obtained at all temperatures and polymer fractions. Due to the spontaneous formation of the bilayers, there was a slight discrepancy in the number of POPC and/or PBD_22_-*b*-PEO_14_ molecules on each leaflet (≤3%). Since the observed difference was minor and probably caused by random events during self-assembly, we employed a Matlab 2010 code to adjust the molecular distribution and achieve a fully symmetric bilayer after the initial equilibration. This approach was used to standardize the simulation results and ensure that the bilayer would not have any residual stress related to uneven distribution between leaflets.^37^

The formed bilayers were under non-zero stress due to the isotropic pressure coupling scheme. To address this, semi-isotropic pressure coupling was applied in the sequence with a further 100 ns equilibration. Runs for data collection were performed for at least 10 μs with a 20 fs time step, and the absence of significant variations in the properties for at least 3 μs was considered the convergence criterion. Particle trajectories were analyzed using the Visual Molecular Dynamics (VMD) software,^38^ Gromacs built-in tools, Martini scripts available on its official website, and in-house codes. The systems were characterized in terms of membrane thickness, lateral area, mass diffusivity, order parameter of the POPC acyl chains, electron density profiles (EDPs), pressure profiles, Gaussian curvature modulus, bending modulus, and area compressibility modulus.

The lipid/polymer lateral area was calculated by dividing the lateral area of the simulation box by the number of molecules per leaflet. The mass diffusivity was calculated by a linear regression of the mean square displacement according to Einstein’s relation. The average thickness of the bilayer was obtained by subtracting the volume occupied by the water from the total volume of the simulation box and dividing the result by the area of the box parallel to the bilayer plane (*h* = (*V*_box_ – *V*_water_)/*A*_*xy*_), as done in ref (39). For pressure and EDPs, the center of mass of the bilayer was positioned at the center of the *z*-axis (*z* = 0). The system was divided into 200 slices, and the properties of each slice were averaged over the trajectory. Pressure profiles were obtained by subtracting the normal and lateral components of the stress tensor (τ_0_ = τ_L_ – τ_N_) using the modified version of Gromacs proposed by Vanegas and co-workers^40^ (Gromacs-LS). The order parameter (*S*_cd_) is a useful measurement of the fluidity of phospholipids and reflects the orientation of the acyl chains with respect to the normal direction of the bilayer. Since the fluidity of phospholipids is primarily driven by an increase in the entropy of the acyl chains, the *S*_cd_ of hydrophobic beads was averaged to provide a global characterization of this variable. The order parameter is calculated as *S*_cd_ = 0.5⟨3 cos^2^ θ – 1⟩, where ⟨⟩ denotes the ensemble average, and θ is the angle between the bilayer plane (*z*-axis) and the distance vector between two bonded beads in the lipid side chains.

The area compressibility modulus (*k*_A_) was calculated from the amplitude of fluctuations in the lateral area of the simulation box by applying the following relation
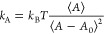
1where *k*_B_ is the Boltzmann constant, *T* is the temperature, *A* is the lateral averaged area of the bilayer, and *A*_0_ is the instantaneous area.

The Gaussian curvature modulus (κ_G_) was obtained using the stress profile method described in ref (41). In the case of null membrane tension, the integral of the stress profile along the *z*-coordinate is zero . If this condition is satisfied, the Gaussian curvature modulus can be obtained by the second moment of the lateral stress profile, as shown in eq 2
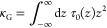
2

The bending modulus (*k*_C_) was determined using the buckling method described in ref (42), which was developed considering a variety of membrane compositions and is applied here in the context of HVs. For this purpose, larger membranes (consisting of 1000 molecules) were assembled in a 32 × 8 × 20 nm^3^ simulation box containing 35,516 water molecules. The reason for using a larger system was to address convergence issues that were previously reported when applying this method to smaller membranes.^43^ After initial equilibration, the system was subjected to compression in the *x*-axis while maintaining a constant *y*-dimension until a strain of λ = (*L*_0_ – *L*_compressed_)/*L*_0_ = 0.2 was reached. Then, simulations were performed with the stressed membrane for 8 μs with restrained *x* and *y* dimensions, and the force required to maintain the membrane’s compressed shape is related to *k*_C_ by eq 3
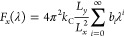
3where *F*_*x*_ is the force exerted in the buckled membrane (calculated by *F*_*x*_ = *P*_*xx*_*L*_*y*_*L*_*z*_), *L*_*y*_ and *L*_*x*_ are the membrane dimensions in the *y* and *x* axis, and *b*_*i*_ are the coefficients given in the original paper by Diggins and Deserno.^42^ The infinite sum represents an analytical solution obtained from a series expansion of the Helfrich Hamiltonian, which is used to analyze the bending modulus of the membrane. To facilitate computation, the sum is truncated at the 10th term (*i* = 10). The effects of thermal fluctuations were included in the overall force by the addition of a term σ*F* = −1.5*k*_B_*T*∑_*i*=0_^∞^*d*_*i*_λ^*i*^/*L*_*x*_, where σ*F* is the contribution of thermal undulations to the force, and *d*_*i*_ are the coefficients published in ref (42).

### Analysis of the Interactions between HVs and a WALP Peptide

As one of the advantages of HVs compared to pure polymersomes is the functional incorporation of proteins, simulations of a WALP peptide incorporated into the membrane were performed. WALP is a helical peptide composed of glycine, tryptophan, alanine, and leucine that is widely used in computational biology as a model for transmembrane proteins.^29,44,45^ The dynamics of WALP in bilayers can be accurately described by Martini 3, as shown before,^45^ provided that the peptide length is not too short (≤12 amino acids). Considering this and the fact that transmembrane α-helices usually span between 17 and 25 amino acids,^46^ the peptide WALP_23_ was used.

The CG model of WALP_23_ was generated as follows: an AA representation was created using the Avogadro software, with each residue assumed to adopt an α-helical conformation. Next, Martini beads with bonded parameters were assigned to each amino acid using the Insane python script.^47^ The peptide was then aligned and inserted into a pre-equilibrated membrane, and the system was subjected to energy minimization using the steepest descent algorithm. To examine the interaction of the peptide with membranes of varied compositions, umbrella sampling (US) simulations were carried out to calculate the potential of mean force (PMF). US simulations have already been conducted to analyze the dynamics of various peptides in lipid membranes^29,44^ and are used in this study to assess the change in free energy that occurs when the peptide is pulled through HVs with different lipid/polymer compositions. Multiple configurations were generated, with the peptide gradually moving in increments of 0.1 nm along the *z*-axis toward the outer side of the bilayer until it was fully immersed in water and no longer interacting with the membrane. A representation of the system and the histogram of the umbrella sampling windows can be seen in Figure S7 of the Supporting Information. The overlap between adjacent windows suggests that the simulations were converged and properly spaced.

It is possible to pull the peptide from the membrane starting at both its carboxyl and amine termini, with slightly different energy barriers expected in each case.^48^ Here, results starting from the N terminus are reported, but the same analysis was conducted for the C terminus, which is discussed in Section S2A of the Supporting Information. For each initial configuration, we performed an energy minimization followed by a 50 ns equilibration on the isobaric-isothermal ensemble (*NPT*, *P* = 1 atm, *T* = 300 K). The position of the peptide was restrained by applying a harmonic potential with an energy constant of 1000 kJ mol^–1^ nm^–2^. Data acquisition simulations were performed for 200 ns for each window. The PMFs were calculated using the weighted histogram analysis method (WHAM),^49^ as implemented in the Gromacs tool *gmx wham*. The results were found to be consistent with equilibrium simulations by performing additional runs without restrictions on the peptide. The final position of the molecule was in agreement with the point of minimum energy in the PMF analysis for all cases.

Systems that showed an energy minimum when the peptide was in a transmembrane configuration were further examined in 5 μs simulations, which were conducted without restriction potentials for all four temperatures described previously. 2D profiles were analyzed to assess the local impact of peptide insertion on different membrane compositions. The thickness and density of the membrane, as well as the order parameter of the lipid acyl chains, were calculated. Profiles were obtained by discretizing the membrane in a 0.5 nm grid along the *x* and *y* directions. To ensure that no empty cells were present, the non-local weighted-averaging method in ref (50) was employed, as described in Section S2B of the Supporting Information.

## Experimental Analysis

### Hybrid Vesicle Formation

The protocol for preparing pure and HVs is similar to the previously published method of ref (17) and is briefly described here. To create vesicles with different compositions, the appropriate volume of 6.6 mM PBD_22_-*b*-PEO_14_ (Polymer Source, Inc.) CHCl_3_ suspension was mixed with 33 mM POPC (Avanti Polar Lipids) in CHCl_3_:MeOH in a glass vial. The mixture was dried in a vacuum desiccator for 4 h to form a multilayer film, which was rehydrated with a 20 mM HEPES buffer (Thermo Scientific, pH 7) containing 50 mM of the fluorescent dye 5(6)-carboxyfluorescein (Novabiochem). Heating cycles of 50 °C for 5 min followed by vortexing for 1 min were performed until the films completely dissolved in the buffer. The resulting suspension was frozen in liquid nitrogen, heated to 60 °C, and shaken vigorously for 15 s; this cycle was repeated five times.

The aliquots were extruded 11 times through a 100 nm polycarbonate membrane filter using an Avanti Mini extruder, resulting in vesicles of reproducible size. Extrusion was performed at 60 °C to facilitate the sample extraction. The formation of vesicles was confirmed by dynamic light scattering measurements (DynaPro NanoStar). The particle size distributions, as shown in Figure S14 of the Supporting Information, indicate that the average vesicle size is approximately 100 nm, which is in line with the size of the filter used.

### Fluorescence Spectroscopy

The vesicles were formed in an aqueous solution containing carboxyfluorescein, resulting in the encapsulation of dye molecules. The mechanical stability of these structures can then be evaluated by measuring the increase in fluorescence, caused by the release of the dye during the disruption of vesicles upon addition of a surfactant. A previous study conducted similar investigations on HVs, focusing on the spontaneous disruption that occurs over time.^51^ In contrast, the present study addresses the disruption kinetics by introducing a detergent to catalyze the destabilization of the vesicles. The excess carboxyfluorescein outside the vesicles was removed from the solution through size exclusion chromatography using a NAP-5 column (Cytiva) according to the manufacturer’s instructions. The suspensions were then diluted 100-fold and exposed to varying concentrations of Triton X-100 (Sigma-Aldrich). Fluorescence spectroscopy measurements were conducted using a FLUOstar Omega microplate reader with excitation and emission wavelengths of 485 and 520 nm, respectively. Normalized curves were obtained by setting the fluorescence (*F*) of the original samples to 0, and considering a complete disruption achieved by adding 10% Triton (m/v) to the vesicle suspension as having a fluorescence value of 1. This disruption assay was performed at two temperatures (298 and 318 K). The normalized data were fitted to a fourth-party logistic model (4PL), represented by the equation: *F* = *d* + (*a* – *d*)/[1 + ([*T*]/*c*)^*b*^]. In the equation, *a* is the theoretical response at zero concentration, *b* is the slope factor, *c* is the mid-range concentration (inflection point), and *d* is the theoretical response at infinite concentration. [*T*] is the Triton X-100 concentration in % (m/v). Since the data was already normalized, the values of *a* and *d* were fixed to 0 and 1, respectively, while the remaining parameters were estimated using a Matlab code.

## Results and Discussion

### Structural Properties

**Figure 2:**
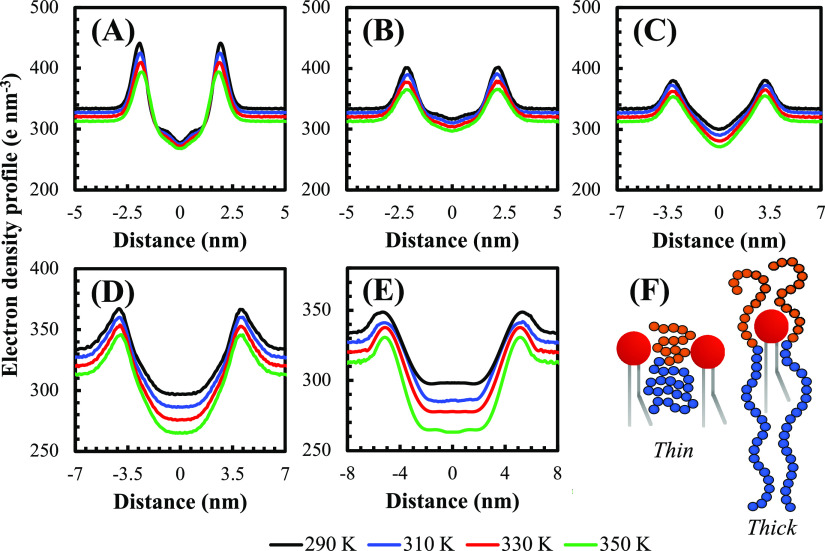
EDPs as a function of distance from the bilayer center of mass obtained from MD simulations. (A) Pure liposome, (B) 25% polymer:75% phospholipid HV, (C) 50:50 HV, (D) 75:25 HV, and (E) pure polymersome. Proposed configurations for the thick and thin regions are shown in (F), where red, orange, and blue beads represent phospholipid headgroups, PEO monomers, and PBD monomers, respectively. The lines correspond to the lipid acyl chain.

Recently, the EDPs of HVs composed of POPC and PBD_22_-*b*-PEO_14_ were determined using SAXS and cryo-electron tomography techniques.^22^ To validate the simulation results, the EDPs at different temperatures were compared with the experimental data. The density profiles of both leaflets were found to be symmetrical and in agreement with the experiments, as depicted in Figure 2. Minor asymmetries were reported and attributed to curvature effects at the membrane;^22^ as the simulation profiles are based on planar bilayers, this behavior was not seen, supporting the hypothesis of the authors. As the polymer fraction increases, the peak-to-peak distance becomes longer, indicating a larger membrane thickness. The peak-to-peak distance of a POPC membrane was reported to be 36.4 Å in SAXS experiments, while a cryo-electron microscopy analysis using a fast Fourier transform (cryo-FFT) showed a distance of 35 Å, in very good agreement with the value of 36.9 Å predicted in the simulations. Particular attention can be paid to the pure polymer membrane, as the present study aims to provide a reliable CG model for PBD-*b*-PEO. In this case, both SAXS and cryo-FFT reported a peak-to-peak distance of 108 Å, which is in close agreement with the simulation prediction of 106 Å.

**Figure 3:**
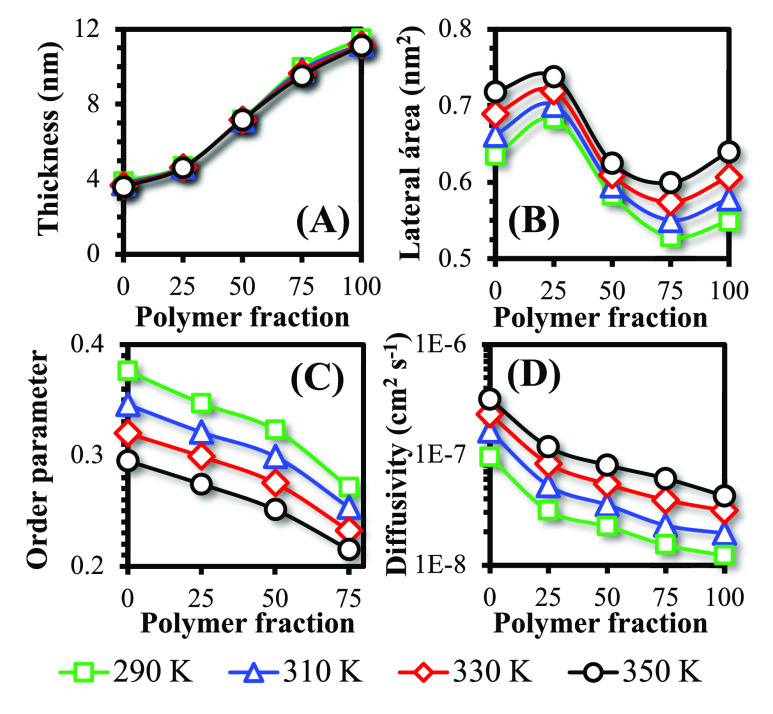
HVs (A) thickness, (B) lateral area, (C) lipid order parameter, and (D) mass diffusivity as a function of polymer composition for varied temperatures, computed from the simulations.

Experimental observations have shown the existence of two distinct populations of 50:50 HVs with different membrane thicknesses.^22^ One of these has an EDP similar to that of polymersomes (“thick” membrane), while the other resembles that of pure liposomes (“thin” membrane). This is an interesting observation given that earlier fluorescence studies have shown that POPC and PBD_22_-*b*-PEO_14_ form well mixed and homogeneous vesicles containing both polymers and lipids in their composition.^26^ We previously hypothesized that the coexistence of these two distinct membrane structures in otherwise homogeneously mixed membranes indicates that the thin and thick structures have comparable free energies, while intermediate structures are energetically unfavorable.^22^ The CG representation in the present study appears to accurately model the two distinct populations of HVs. Both lipid-only membranes and the 25% polymer:75% phospholipid HVs showed qualitatively similar EDPs and thin membranes (Figures 2A,B and 3A). The same is true for the 75:25 HVs and pure polymersomes (Figure 2D,E), which both show thick membranes. In the 50:50 simulations, the average membrane thickness falls between those of the two observed conformations (Figures 2C and 3A), but closer inspection shows the coexistence of thicker and thinner regions (Figure 1). The co-existence of both phases for this particular composition was previously reported,^22^ which supports the accuracy of the present CG parametrization to model HVs. While the existence of two different conformations is not readily visible in the average EDPs shown in Figure 2C, Figure S16 of the Supporting Information shows separated profiles for each component in the system. This analysis reveals the presence of acyl chains both at the center and at the extremities of the bilayer, providing additional evidence of two configurations in the simulations. Further evidence is also presented in Figure S18 of the Supporting Information, which depicts the spatial distribution of the phosphate–phosphate distance between the leaflets for a 50:50 HV. The plot in Figure S18 shows distinct regions with a thickness comparable to that of POPC (≈40 Å) surrounded by domains of thicker conformations (≈70 Å). On average, approximately 20% of the membrane was in the thin configuration (with a thickness of 50 Å or less). Experiments^52^ and AA MD simulations^24^ also revealed the presence of different conformations related to changes in the copolymer chain lengths and concentration.

In an effort to understand the nature and origin of these different bilayer conformations, a more detailed description of the regions with thinner and thicker conformations is presented. The bottom part of the membrane shown in Figure 1 displays a thinner conformation, where the phospholipids in opposite leaflets are closely packed, as observed in pure lipid membranes. The elastic chains of polymers are able to adjust to this configuration by becoming more entangled and bending toward the lateral regions, as depicted in Figure 2F. Moreover, the apolar part of the polymers tends to interdigitate more tightly in this region to conform to its intricate topographical features, as shown in Section S7 of the Supporting Information. The polar part of the copolymer adopts a mushroom-like structure to conform to the choline and phosphate groups of the phospholipid head group. In general, mushroom-like structures are formed at low polymer densities,^53^ and therefore, the thin region may result from a slightly lower local polymer concentration in the bilayer. Figure 3B supports this conclusion by showing that the lateral areas of lipid/polymer mixtures increase in 25:75 HVs, which is expected when a collapsed structure is present. In the case of the thicker conformation at the top part of the membrane depicted in Figure 1, a locally high polymer density results in an extended polymeric structure, with a more elongated configuration for both the polar and apolar units (as also seen in Figure 2F). The phospholipids in both leaflets are then located at the hydrophobic–hydrophilic interface of the copolymer, with minimal interaction between the phospholipids in the opposite leaflets. This behavior can be visualized through qualitative analysis of the PEO peak widths in EDPs. Specifically, systems with elongated configurations exhibit greater peak width (≈20 Å, in comparison with ≈12 Å for the thin conformation), as evidenced by Figure S16 of the Supporting Information.

It is worth noting that our previous study did not find different membrane configurations within the same vesicle, but vesicles exhibiting either thicker or thinner conformations.^22^ In our simulations, we observed both conformations within the same membrane for 50:50 HVs. This deviation can be understood by a more in-depth analysis of the lipid/polymer concentration at each phase. In the thinner region, the polymer–lipid ratio was slightly lower (at around 40% polymer and 60% lipid), causing the polymer to conform to the lipid structure. This conformation was caused by the polymer adopting a more entangled structure and a higher degree of interdigitation, as discussed in the previous paragraph. On the other hand, when the polymer concentration was slightly higher, the phospholipid adapted to the elongated structure of the polymer. This observation suggests that, even though the vesicle preparation in experiments uses a 50:50 ratio, slightly different compositions between individual vesicles might drive the complete vesicle toward either a thin or thick conformation. In our simulations, we used an exact 50:50 proportion, and therefore, the different configurations resulted from local fluctuations in the polymer–lipid concentration within a region of the same membrane.

The average lateral area, membrane thickness, order parameters of the acyl chains, and lateral diffusion coefficient at various temperatures and membrane compositions obtained from the simulations are presented in Figure 3. Small-angle neutron scattering experiments measured the average lateral areas of POPC, which ranged from 0.627 to 0.681 ± 0.013 nm^2^ for a temperature range of 293–333 K.^54^ These results agree well with the present simulations, which predicted values between 0.635 and 0.689 nm^2^ for similar temperature conditions (290 to 330 K), as shown in Figure 3B. Varied estimates for this variable have been reported for copolymers, depending on the level of analysis and whether experiments or simulations were analyzed. For a slightly longer chain (PBD_23_-*b*-PEO_16_), cryo-TEM measurements^55^ reported a lateral area of 0.65 ± 0.05 nm^2^. The same study also proposed a CG model for this slightly longer molecule, which predicted a lateral area of 0.77 nm^2^ at 323 K. An AA model was proposed for PBD_22_-*b*-PEO_14_ using the CHARMM FF and found an area of 0.87 nm^2^.^24^ The results of the present study are lower than those reported in previous simulation studies but are in better agreement with cryo-TEM results.^55^ Bermudez and co-workers developed a mathematical model based on experimental measurements to estimate the lateral area of diblock copolymers as a function of the molecular weight of the hydrophobic block.^56^ Using the parameters for hydrophobic chains with intermediate to high molecular masses (>1 kg mol^–1^), the expected area of PBD_22_-*b*-PEO_14_ is 0.63 nm^2^, in agreement with the cryo-TEM measurements. The presented results agree well with these experimental observations and tend to slightly underestimate them by ≈6%.

Although the lateral area of the pure polymer membrane is smaller than that of the pure phospholipid, the addition of a small amount of polymers (25%) to a POPC membrane leads to an increase in this area. This supports the hypothesis of a collapsed polymer structure at low polymer concentrations, as discussed earlier. Moreover, the lateral area exhibits a similar qualitative trend to the experimentally-obtained permeability profile of these vesicles (permeability increases for 25:75 HVs and decreases at higher polymer concentrations).^17^ Previous research on phospholipids has established a correlation between permeability and lateral area. Specifically, membranes with a higher lateral area exhibit higher permeability due to the formation of local defects such as gaps or disordered regions.^57,58^ These defects can serve as pathways for molecules or ions to cross the lipid bilayer, ultimately resulting in higher permeability. Given these findings and the aforementioned similar trend between our results and previous experimental reports,^17^ we can infer that the same relationship holds for HVs. In addition, the lateral area increased with temperature, likely due to the increased mobility of the molecules, which reduces the packing density of the membrane and increases its lateral area.

The order parameter of phospholipids is shown in Figure 3C, and an interesting trend is found: as the polymer content increases, *S*_cd_ decreases, indicating that the entropy of the acyl chains of phospholipids rises at higher polymer concentrations. In addition, *S*_cd_ offers information about the state of the lipids, with lower and higher values indicating a more fluid or gel-like state, respectively. This observation may help explain some previous experimental reports related to the incorporation of membrane proteins into HVs, specifically with respect to the more amenable reconstitution of membrane proteins into these structures.^18,19^ Given that increased fluidity can increase the permeability of membranes,^59^ the elevated entropy of the side chains may be a plausible explanation for this phenomenon. It is worth noting that *S*_cd_ applies only to the lipids and has no broader effect throughout the entire membrane. Diblock copolymers often exhibit high viscosity,^60^ which may lead to a general increase in membrane adhesion forces, as confirmed by fluorescence anisotropy measurements.^19^ Furthermore, an increase in temperature causes a corresponding increase in the entropy of the acyl chains, reducing the order parameter and increasing membrane fluidity.

Figure 3D shows the average lateral mass diffusion coefficient of the membranes at different temperatures. In Figure S15 of the Supporting Information, we provide lateral mass diffusion coefficients for both lipids and polymers separately, which agree with our previous fluorescence recovery after photobleaching measurements,^26^ which demonstrated a decrease in diffusion with increased polymer content. The simulations show that the overall diffusion coefficient of the membrane increases significantly with temperature while decreasing with polymer content. This indicates that indeed a decrease in fluidity is observed in membranes with higher polymer concentrations, as suggested in the previous paragraph. Additionally, fluorescence experiments revealed that HVs of POPC and a polymer with a longer chain (PBD_48_-*b*-PEO_30_) also exhibited decreased fluidity as the polymer content increases.^27^ This observation suggests that the addition of this type of block copolymer may generally reduce membrane fluidity. The individual diffusivity of POPC in the simulations is higher than that of PBD-*b*-PEO, which is expected due to the reduced free volume required for lipids to hop laterally between sites in the membrane matrix.^26^ Moreover, higher temperatures lead to more fluid membranes as a result of decreased viscosity and increased molecular mobility.^61^ The simulated values are in good agreement with the diffusivity range measured experimentally for pure POPC membranes at room temperature using nuclear magnetic resonance (3.2 × 10^–8^–1.9 × 10^–7^ cm^2^ s^–1^).^62,63^

The results of this subsection helped shed some light on HV-related phenomena, such as the reasons behind the presence of two distinct membrane conformations despite the absence of clear phase separation between polymers and lipids. Additionally, the structural properties predicted by the proposed model are in very good agreement with previous experimental results for both pure liposomes/polymersomes and HVs. The dependence of several of these properties on the polymer content potentially enables tuning the membrane environment for several practical applications of HVs, as discussed elsewhere for drug delivery and synthetic biology.^64−66^ One of the most noteworthy benefits of incorporating polymers in lipid membranes is the enhancement of vesicle robustness, which is closely tied to their mechanical properties and are addressed in the next section.

### Mechanical Behavior

**Figure 4:**
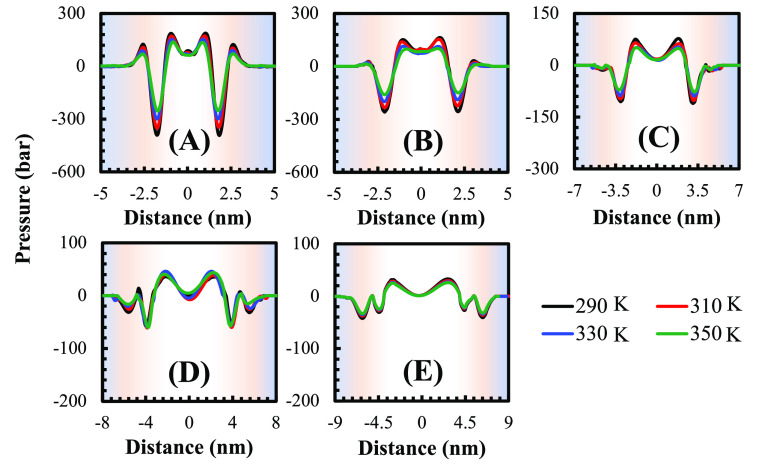
Lateral pressure profile as a function of the distance to the bilayer center of mass at varied temperatures for (A) pure liposome, (B) 25 polymer:75 phospholipid HV, (C) 50:50 HV, (D) 75:25 HV, and (E) pure polymersome. The colored regions correspond to the water (extremities), hydrophilic region, and hydrophobic core (center).

The lateral pressure profiles of the simulated bilayers under all conditions studied are presented in Figure 4, providing valuable insight into membrane interactions by revealing the relationship between stress and strain on these structures. The pressure in all curves approaches zero at the edges of the simulation box, demonstrating that the systems are fully hydrated. Positive and negative peaks indicate areas where expansion and contraction forces are dominant, respectively. Importantly, these forces decrease with increasing polymer content, suggesting that polymer-based membranes are subjected to lower local stresses.

In lipid bilayers, the two positive peaks at the extremities are associated with the polar head-groups, where repulsive electrostatic interactions and steric effects at the membrane–water interface generate an expansion force at the bilayer. There is also a positive peak in the hydrophobic core region, which is slightly decreased in the center of the membrane where cohesion between lipids takes place. To counterbalance these repulsive forces, the glycerol groups at the hydrophilic/hydrophobic interface exert an attractive compressive force. These results are consistent with profiles reported previously for POPC membranes.^67,68^ The first moment of the stress profile  is close to zero, as shown in Table S2 of the Supporting Information, indicating that the compressive and expansive forces are in balance, as expected for stress-free bilayers.

The pressure profiles observed for HVs show a dependence on the polymer-to-lipid ratio. At 25:75, the profile is similar but smoother when compared to pure lipid bilayers, suggesting a reduction in the intensity of local forces as polymers are incorporated into the membrane. For 50:50 HVs, a negative peak starts to appear at the edges of the bilayer, followed by a decrease in the positive peak related to the hydrophobic core. At a 75:25 ratio, this behavior is even more pronounced. These results suggest that PEO contributes to the cohesion of the bilayer, replacing the compression forces previously exerted by the glycerol groups. Indeed, in pure polymer membranes, there is no positive peak in the hydrophilic core, but only two negative peaks, indicating that the attraction force is caused mainly by the hydrophilic chain and its interactions with water (first negative peak) and the hydrophilic core (second negative peak). As for the effects of temperature, a slight decrease in the stress profiles is observed for the same composition as *T* increases, suggesting that the mechanisms described above are similar at different *T* conditions. Similar qualitative/quantitative profiles for polymer membranes have also been reported before.^69^

**Figure 5:**
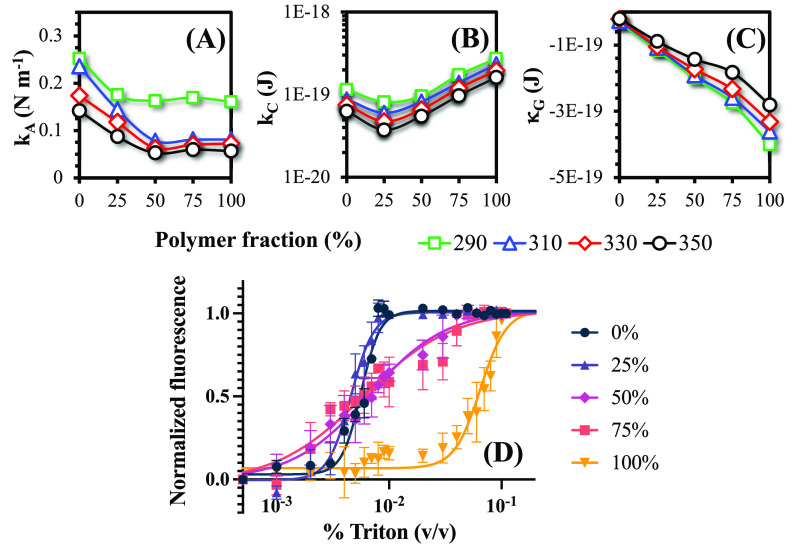
HVs (A) area compression, (B) bending, and (C) Gaussian curvature modulus as function of polymer composition for varied temperatures, as computed from the simulations; (D) Triton X-100 destabilization profile at 25 °C for varied polymer concentrations measured in the experiments.

The pressure profiles obtained for HVs have implications for the insertion of membrane proteins. High pressures in hydrocarbon cores can hinder the insertion of proteins into these environments.^70^ The simulations indicate that an increase in the polymer concentration may reduce the intensity of the repulsion in the hydrophobic core, potentially making it easier for proteins to be incorporated. This aspect will be addressed in the next section. To gain a more comprehensive understanding of the mechanical properties of HVs, we combined simulations with experiments to conduct additional analyses. The results are presented in Figure 5, which illustrates the elastic moduli obtained from simulations as well as the mechanical stability of the vesicles following the addition of Triton X-100 in experiments.

It can be seen that the area compressibility modulus *k*_A_ tends to decrease with increasing polymer content, indicating that lower stresses are needed to expand or compress the bilayer, in line with the obtained pressure profiles. It is worth noting that the values for 50:50 and 75:25 HVs and polymersomes were almost identical, suggesting that this modulus can be significantly reduced by just adding 50% polymers to a lipid bilayer. This reduction of *k*_A_ (along with the increased POPC side-chain entropy and fluidity reported in the previous section) can contribute to the easier insertion of peptides in HVs compared to pure phospholipids. This hypothesis was previously suggested by Jacobs and co-workers, who experimentally determined a *k*_A_ = 0.103 ± 0.005 J m^–2^ for PBD-*b*-PEO at room temperature,^19^ which is in good agreement with the averaged value obtained in the simulations on the 290–330 K range of 0.105 J m^–2^ (error = 1.9%). Furthermore, a decrease in *k*_A_ for POPC/PBD_44_-*b*-PEO_30_ HVs was previously reported,^27^ which further supports the simulation results. Higher temperatures resulted in lower *k*_A_ values, which is consistent with experimental observations and is related to the increased thermal fluctuations of bilayers at higher *T*, as predicted by statistical thermodynamics theory.^71^ Moreover, experimental values for pure lipid bilayers range from 0.15 to 0.25 J m^–2^, with a value of 0.21 J m^–2^ for POPC,^72^ which is in good agreement with the average value of 0.2 J m^–2^ from our simulations.

The bending modulus *k*_C_ is a crucial mechanical parameter of lipid bilayers as it quantifies the energy required to deform the membrane from its natural curvature to a different shape. The results are shown in Figure 5B, and, in general, as the polymer fraction increases, the bending modulus increases as well. However, an exception is observed in the 25:75 case, where a decrease is observed instead. Generally, experiments indicate that a higher polymeric content results in vesicles with higher stiffness,^73^ and hence, a higher *k*_C_ is expected in these structures, in line with our simulation results. The deviant behavior in the 25:75 case may be due to the increase in lateral area under these conditions (as shown in Figure 3B). In general, larger bilayers are more flexible and easier to bend,^74^ so the slight decrease in *k*_C_ in this case may be attributed to the higher surface area in these conditions. Regarding temperature effects, a higher *T* increases the thermal energy of the system, causing the molecules in the bilayer to become more disordered and undergo greater thermal fluctuations. This effect makes the membrane more easily deformable and reduces the bending modulus of the bilayer.^75^ This behavior was observed in all lipid/polymer mixtures studied.

The Gaussian curvature modulus κ_G_ describes the energy cost of changing the Gaussian curvature of a surface while preserving its mean curvature, in contrast to *k*_C_, which describes the energy cost of deforming a surface to change its mean curvature. Increasing the polymer content and temperature leads to a lower Gaussian curvature modulus, as shown in Figure 5C. The negative values obtained indicate that, for all the lipid compositions studied, the formation of vesicles is favored. Conversely, positive values indicate that other conformations, such as tubular or inverted hexagonal structures, are preferred (as opposed to vesicles). The elastic ratio *k*_C_/κ_G_ is a useful parameter for characterizing how easily the structures of those vesicles can be changed, as it combines information from both the bending and Gaussian moduli. Stable vesicles have elastic ratios below 0,^76^ which is observed in all cases. However, the values of the elastic ratios vary between ≈−0.3 for pure POPC and ≈−1.6 for pure polymer vesicles. This suggests that although vesicular structures are thermodynamically favored in both cases (elastic ratio ≤ 0), pure POPC membranes are more susceptible to destabilization (formation of micelles or disorganized structures).

While these elastic moduli and their relationships provide valuable insights into the mechanical properties of the bilayers, they do not directly translate into quantitative assessments of the mechanical stability. Assessing stability through molecular dynamics simulations is challenging due to limitations in the simulation time scales (up to ns−μs). Lim and co-workers^28^ used fluorescence experiments to investigate the disruption of vesicles caused by naturally occurring pores on the membrane surface. They found that the addition of polymers significantly delayed the natural release of dye, and in the case of pure polymersomes, the release was less than 10% even after five days. However, it is unclear whether this stability persists under adverse conditions. Based on the previous discussion of the elastic ratio, it can be predicted that polymer-rich membranes will lead to more stable vesicles even under increased mechanical stress.

To confirm this hypothesis, we conducted fluorescent experiments on vesicle stability, exposing them to different detergent concentrations at two temperatures (298 and 323 K). The results at 298 K are presented in Figure 5D. Overall, the results of the detergent destabilization experiments corroborated an important characteristic of HVs: improved mechanical stability compared to pure liposomes. For pure liposomes, when the detergent concentration reached 0.003% (v/v), the encapsulated carboxyfluorescein started to be released, and at a concentration of 0.009%, the encapsulated dye was already fully released. Similar results were obtained at 45 °C, although the fluorescence reached its maximum value more quickly (*C* = 0.008%), indicating that the kinetics of lipid destabilization is accelerated at higher temperatures. We observed a similar behavior in 25:75 HVs, and a comparison of the 4PL model parameters provided in Table S3 of the Supporting Information showed that the kinetic behavior in both cases is indeed similar.

At higher polymer concentrations, the destabilization rate was considerably slower, with complete leakage occurring only at detergent concentrations of 0.05 and 0.07% for 50:50 and 75:25 compositions, respectively. This indicates a nearly tenfold increase in detergent concentration necessary to completely disrupt vesicles with a higher polymer concentration. This can also be seen by the logistic model parameter *b* on Table S3 of the Supporting Information, which shows that a higher Triton concentration is required to achieve 50% of the maximum leakage in polymer-rich HVs compared to pure liposomes and 25:75 HVs. Furthermore, the lower *c* parameter observed for the 50:50 and 75:25 vesicles, compared to pure liposomes, suggests slower disruption kinetics in these cases. These findings are in agreement with a previous study by Nam and co-workers,^51^ which reported that the addition of detergent primarily affects lipid-rich domains, but leakage is prevented by the presence of surrounding polymer molecules that cover the resulting pores. For pure polymersomes, complete disruption was achieved at a detergent concentration of 0.09%, but leakage only began when *C*_Triton_ = 0.01%. These results indicate a substantial increase in vesicle stability upon the addition of PBD_22_-*b*-PEO_14_, which is in good agreement with the aforementioned simulation results of lower elastic ratios in such conditions.

In conclusion, the addition of polymers has an impact on all measured mechanical properties. Lateral pressure profiles and the area compression-expansion modulus from the simulations indicate that the forces required to stretch the membrane are significantly lower in polymer-rich vesicles. The combined effect of the bending and Gaussian curvature moduli indicates that polymer-rich membranes result in more stable vesicles, as confirmed by the fluorescence leakage experiments upon addition of detergent. Therefore, as noted in several publications,^13,17−19,22,24,26^ HVs are robust structures that can be used as effective alternatives to pure polymersomes and liposomes. This set of mechanical parameters shows that HVs have a potential advantage over pure liposomes/polymersomes when applied to the release of encapsulated compounds. In the context of drug delivery, it is crucial for vesicles to withstand the high osmotic pressure and shear flow in blood vessels without leakage. This property can be finely tuned by controlling the polymer/lipid ratio.^77^ While lipids tend to destabilize rapidly, polymersomes are often too stable and do not provide a sufficiently quick release.^2^ The calculated moduli and detergent stabilization profiles highlight the potential of HVs to modulate drug release, as supported by the Reimhult and Virk literature review.^2^ To explore the interaction of HV with membrane proteins, the following section will focus on the dynamics of pure and HVs with the WALP_23_ peptide.

### Incorporation of WALP_23_ Peptide

**Figure 6:**
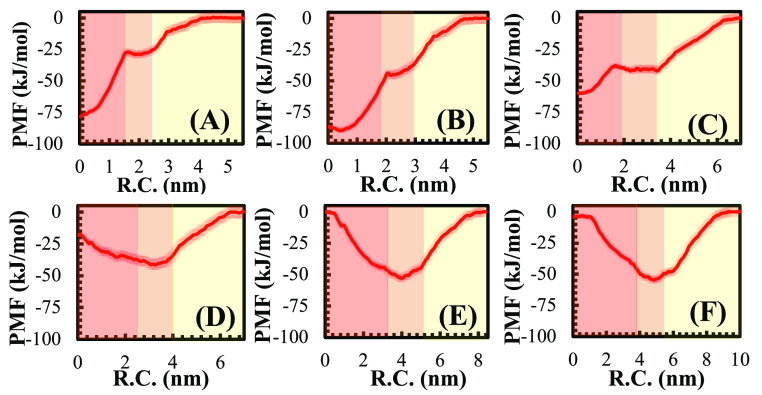
PMF as a function of the reaction coordinate for (A) pure liposomes, (B) 25:75 HV, (C) 50:50 HV with the peptide in the thin region, (D) 50:50 HV with the peptide in the thick region, (E) 75:25 HV, and (F) pure polymersome. The colored regions, from left to right, correspond to the hydrophobic core, hydrophilic region, and water, respectively.

The free energy profiles of the WALP_23_ peptide as it is extracted from the bilayer starting from the N-terminus (computed from US simulations) are depicted in Figure 6. As the free energy of solvation of the peptide in water is equal for all membranes, we chose the aqueous region that is far from the membrane’s surface as a reference point. In other words, this region serves as a baseline, where the PMF value is equal to 0 kJ mol^–1^.

For the 50:50 HVs, two scenarios were analyzed: the peptide starting from either the thin or the thick region (as defined and discussed previously). It is evident that the PMFs vary significantly among different membrane compositions. In pure liposomes and 25:75 HVs, the lowest energy is found at the center of the bilayer, and energy gradually increases as the peptide moves away from the membrane and into the solvent, indicating that the transmembrane orientation of the peptide is thermodynamically favored, as previously reported.^45,48^ As the peptide moves away from the center, energy increases due to the increased repulsive interaction between the hydrophobic helix core and the membrane hydrophilic region. At the same time, the C-terminus is moved into the hydrophobic core of the bilayer, also contributing to the energy increase. When the peptide reaches the hydrophilic area of the membrane, it aligns along the *x*–*y* plane to minimize surface contact between its charged ends and the acyl chains of the bilayer, and in this region, the energy remains almost constant. After the peptide is fully removed from the bilayer, its charged termini are still attracted to the POPC head groups via electrostatic interactions, causing the system’s energy to increase as the peptide moves away from the membrane. Once the peptide–bilayer distance exceeds the electrostatic cutoff (i.e., the peptide is sufficiently far from the membrane surface), the free energy of the system remains constant.

For 50:50 HV bilayers, the behavior of the peptide depends on its starting location (thin or thick region). If it starts within the thin region, a profile similar to that seen for lower polymer fractions is observed (Figure 6C), although the difference in free energies between the transmembrane and fully solvated configurations is slightly smaller, indicating that less energy is required to extract the peptide from the membrane. Notably, the energy difference between the bulk water and the initial interaction with the membrane hydrophilic part is larger than in the case of pure lipid membranes. This suggests that the initial interaction of the peptide with the protein is more favorable for 50:50 HVs, which could help to explain why proteins insert more readily into HVs than into liposomes, as observed in in vitro reconstitution studies.^18,19^ Conversely, when the peptide starts within the thick region of the 50:50 HVs, a different profile is obtained, with the energy minimum shifted to the hydrophilic part of the membrane. In this case, the charged termini of the peptide cannot reach the hydrophilic regions of the membrane due to the significant size difference between the thickness of the bilayer and the length of the peptide. As the peptide moves closer to the hydrophilic core, the energy decreases and reaches a minimum when the peptide is parallel to the bilayer plane and fully inserted into the hydrophilic region, where electrostatic interactions between the polar components of the membrane and the charged termini of the peptide are minimized. Upon removal from the bilayer, the energy increases again due to the electrostatic attraction to the membrane, as previously mentioned. Similar behavior is also observed for 75:25 HV and pure polymersomes (Figure 6E,F, respectively).

**Figure 7:**
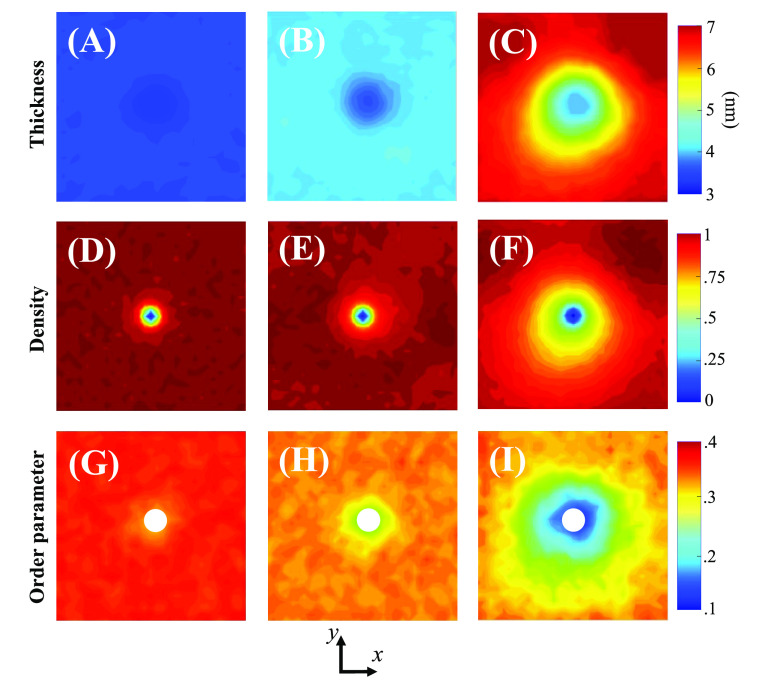
Contour plots showing local measurements of various properties on the membrane’s lateral area as seen in the simulations, including thickness for (A) pure liposomes, (B) 25:75 HV, and (C) 50:50 HV (peptide starting in the thin conformation); membrane normalized density for (D) pure liposomes, (E) 25:75 HV, and (F) 50:50 HV; acyl chain order parameter for (G) pure liposomes, (H) 25:75 HV, and (I) 50:50 HV. The color scale for each variable is displayed in the right-hand side detail.

The PMF results presented demonstrate that the composition of HVs can impact the behavior of peptides in the membrane. We previously reported^13^ that cytochrome *bo*_3_ exhibited higher initial activity when embedded in a pure liposome, with 95 ± 5 and 81 ± 11% of this activity being maintained in 25:75 and 50:50 HVs, respectively. In contrast, only 34 ± 5 and 5 ± 1% of this activity was retained in 75:25 and pure polymersomes. Our simulation results support these experimental findings, as the peptide was found to have an energy minimum in a transmembrane configuration only when the bilayer composition consisted of up to 50% polymer. To further analyze the membrane arrangements near the peptide, Figure 7 provides a visual representation of the membrane thickness, density, and order parameter of phospholipid tails for the mixtures in which a transmembrane peptide was found to be energetically stable (0, 25, and 50% collapsed polymer fraction). The results are displayed as contour plots in the bilayer plane, which is perpendicular to the axis of peptide insertion. To facilitate analysis, the peptide was positioned at the center of the simulation box. The results are presented for a temperature of 310 K, with additional profiles for other temperatures available in Figures S9–S13 of the Supporting Information.

The membrane thickness decreases near the peptide, particularly for the 50:50 HV composition, where a larger decrease is observed. This pronounced behavior in 50:50 membranes is related to the thin and thick phases observed both experimentally and in simulations. We also observe distinct patterns for the density when comparing the different compositions. In the lipid bilayer and 25:75 HVs, the membrane density increases rapidly right after the point where the peptide is inserted. However, the membrane with a 50:50 composition exhibits a larger zone of decreased density. Separate density profiles for the POPC and the polymer can be found in Figures S10 and S11 of the Supporting Information. Comparison of the density profiles for the POPC and the polymer reveals that the decrease in density near the peptide in the 50:50 HV composition corresponds to a higher local concentration of phospholipids and a lower concentration of polymer. These differences do not result in lipid/copolymer phase separation, but they are significant enough to be visualized. This observation supports the hypothesis presented earlier that a local decrease in polymer concentration promotes a collapsed polymeric configuration and reduces the thickness of the bilayer (as seen in Figure 7C). These findings suggest that locally increased phospholipid density may contribute to protein/peptide insertion in HVs, which, together with the thermodynamic analysis by the umbrella sampling technique, may help explain why bioactivity is only preserved at specific polymer-to-lipid ratios.

In addition, Figure 7G–I also suggests a remarkable difference in the distribution of order parameters between pure liposomes and HVs. While the *S*_cd_ in pure liposomes shows a homogeneous and space-independent value which decreases with temperature, as seen in Figure S13 of the Supporting Information, the addition of polymers to the lipid bilayer results in a local shell of decreased order parameter around the peptide. This behavior is observed across all temperatures, as evidenced by the data in the Supporting Information. This provides further evidence that the addition of polymers tends to increase the entropy of the acyl chains of phospholipids, making the hydrophobic core more disordered and favorable for protein incorporation.

The results presented in this study regarding the dynamics of a WALP peptide within various HV formulations have significant implications for practical purposes. The successful integration of proteins into vesicles is crucial for a wide range of biotechnological purposes, such as the development of transmembrane channels for drug delivery,^78^ energy transduction,^79^ and synthetic cell studies.^80^ While these operations are typically carried out using liposomes, our findings align well with prior research on HVs,^13,17,19,20^ suggesting that HVs, with the addition of low to moderate amounts of polymers, can effectively perform similar functions. Furthermore, the reconstitution process in the absence of detergent might be enhanced in hybrid membranes,^18^ facilitating protein incorporation in vesicles.

## Conclusions

In this study, hybrid vesicles (HVs) composed of POPC and PBD_22_-*b*-PEO_14_ were analyzed with computational techniques, and the results contrasted with existing and new experimental data. We evaluated the structural and mechanical properties of these membranes and investigated their biophysical behavior in the presence of a WALP_23_ peptide using coarse-grained MD simulations (with a new parameterization for the copolymer) and the umbrella sampling technique. The simulation results helped shed some light on the different conformations adopted by lipids and copolymers depending on the polymer-to-phospholipid ratio. When the lipid concentration is low, an elongated arrangement is taken by the copolymers, and POPC molecules are positioned at the hydrophilic/hydrophobic interface of the membrane. This leads to increased thickness and decreased lateral area on the membrane. Conversely, at high lipid concentrations, the polymer adapts to the phospholipid configuration by adopting a collapsed structure with decreased thickness and increased lateral area. These structural observations were in good agreement with recent reports about the phase behavior of HVs.

The calculated mechanical modulus showed that the energy required to stretch the membrane is significantly reduced by polymer addition. Furthermore, the addition of polymers tended to increase the fluidity of the POPC acyl chains. These two findings help to explain why proteins are more easily reconstituted in HVs than in pure liposomes. Additionally, the relationship between the bending and Gaussian curvature elastic moduli supports previous findings that HVs become more stable as polymer content increases. This conclusion is also supported by fluorescence experiments that measure leakage in the presence of detergent. Umbrella sampling simulations revealed that the WALP_23_ peptide is only thermodynamically stable in a transmembrane configuration when polymer concentrations are up to 50%, helping to explain why high polymer concentrations may not favor active incorporation of proteins. Our findings are consistent with previous studies and provide new insights into the behavior of HVs that can be helpful in modifying and optimizing these structures for their various biotechnological applications.

## Data Availability

The coordinates of a single polymer chain and its topology, along with an equilibrated bilayer, are available at https://zenodo.org/record/8172733.

## Supporting Information Available

Parametrization procedure, additional umbrella sampling analysis, complete EDPs, and dynamic light scattering experiments (PDF)
