# Supplementary material for: Unraveling the Phase Behavior, Mechanical Stability, and Protein Reconstitution Properties of Polymer–Lipid Hybrid Vesicles
Source: Biomacromolecules. 2023 Aug 4;24(9):4156–69. doi: 10.1021/acs.biomac.3c00498 (PMC10498451; doi:10.1021/acs.biomac.3c00498)
Supplement: Supplementary file 1 — bm3c00498_si_001.pdf [file bm3c00498_si_001.pdf]

Supporting Information for Unraveling the Phase Behavior, Mechanical Stability, and Protein Reconstitution Properties of Polymer-Lipid Hybrid Vesicles

WAGNER A. MÜLLER, PAUL A. BEALES, ANDRÉ R. MUNIZ, LARS J.C. JEUKEN

1. PARAMETERIZATION OF THE POLYMER

The Martini 3 force field lacks an established model for poly(1,2-butadiene) (PBD) and, consequently, for the PBD-*b*-polyethylene glycol (PEO) copolymer. Although Grillo et al.[1] previously analyzed a similar system with this force field, they focused on the linear configuration of PBD, while our study examines the vinyl isomer. We parameterized the selected isomeric conformation following best practices outlined on the Martini website and in relevant publications[2,3] to obtain a coarse-grained (CG) representation of this molecule. The next sections provide a detailed explanation of how we derived the model.

A. Beads choice and non-bonded parameters

Martini particles typically use a 4-1 approach (i.e., four heavy atoms and their bonded hydrogen atoms are represented by a regular *R* bead). For increased resolution, 3-1 small beads *S* and 2-1 tiny beads *T* can also be used. Figure S1 shows the PBD and PEO monomers, each of which can be modeled by a single particle given the number of atoms in each structure. This is a well-established approach to modeling polymers on the CG scale as previously reported[1,4,5,6,7]. Martini 3 provides various particles to select for representing a group of atoms, depending on the chemical properties of the particles represented by each bead. In this study, we first chose the regular C4 bead to represent PBD and the small N3a bead to represent PEO. The C4 bead was selected because it represents nonpolar chains with double bonds, while the SN3a bead was chosen because it represents ether groups. As PEO only has three heavy atoms, a *S* bead was used to model this monomer.

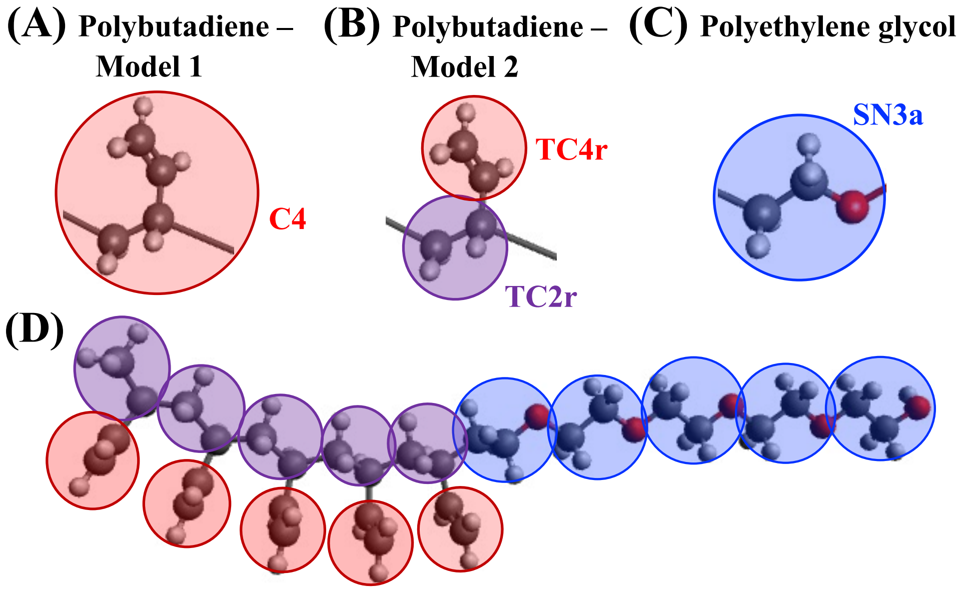

Fig. S1. Molecular and coarse grained representation of (A) PBD model 1, (B) PBD model 2, (C) PEO, and (D) PBD_5-PEO_5.

This standard representation for polymers in CG scale was initially employed, and bonds, angles, and dihedrals for this model were also obtained (as discussed in Section B). This method resulted in a satisfactory description of the PBD-*b*-PEO all-atom (AA) structure, but when polymers and phospholipids were mixed to form hybrid bilayers, we observed a phase separation (Figure S2.A). This outcome conflicted with the expectation of homogeneous dispersion, as shown by Seneviratne et al.[8], indicating that the interactions between the molecules were not accurately computed. To determine which part of the molecule needed to be better modeled, we conducted tests by altering the Lennard-Jones parameters of the C4 and SN3a beads. We found that the C4 beads exerted a considerable repulsion on the acyl chains of the phospholipids, which caused the observed separation.

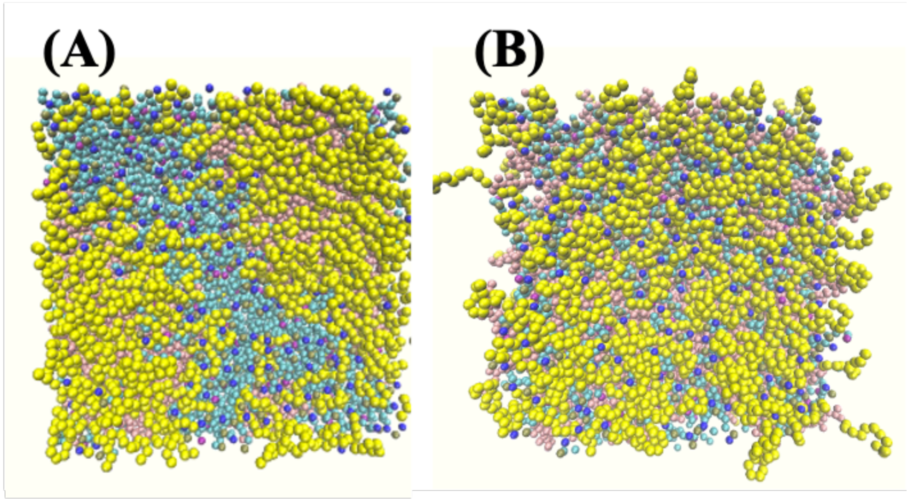

Fig. S2. Hybrid membranes formed by PBD modeled by (A) one and (B) two beads.

To address this issue, a two-bead parameterization using Tiny Martini beads was proposed for PBD. The main chain, which consists of two bonded carbon atoms, was represented by the TC2 bead while the side chain, composed of two double bonded carbons, was represented by TC4 (Polybutadiene - Model 2 in Figure S1). *T* beads were used for this polymer since each particle contains only two heavy atoms. Despite the higher computational cost, this approach produced well-mixed hybrid bilayers (Figure S2.B). Martini beads can be further divided into attractive, normal and repulsive beads, and all different conformations were tested, and electron density profiles were compared with experimentally obtained ones by Ref.[9]. The normal version of the SN3a, along with the repulsive versions of the TC2 and TC4 beads (TC2r and TC4r) were used, which resulted in electron density profiles very similar to the observed in experiments, as discussed in the main manuscript. The developed model also introduced a small amount of hydrophilic PEO beads into the hydrophobic core. This contradicts experimental findings[10], and a similar issue had been previously reported in another study that used the Martini force field to model a linear conformation of the PBD-*b*-PEO polymer[1]. To address this issue, Grillo and co-workers[1] reduced the ϵ parameter describing the interactions between PBD and PEO beads by 11.4 %. Following a similar approach, we decreased this parameter by ≈ 9 %, resulting in $\epsilon_{TC2r-SN3a}$ changing from 1.6 to 1.45 kJ/mol and $\epsilon_{TC4r-SN3a}$ changing from 1.75 to 1.6 kJ/mol. This small adjustment was sufficient to achieve proper separation of hydrophobic and hydrophilic polymer regions.

B. Bonded parameters

The bonded parameters were derived by comparing the distributions of the CG beads with those from all-atom (AA) simulations and further refined to represent experimental features visualized in experiments. PBD and PEO chains with 10 monomers were analyzed to derive parameters for the pure polymers, while PBD_5-*b*-PEO_5 was used to derive parameters for the copolymer hydrophobic-hydrophilic interface. The AA simulations were carried out in the NPT ensemble (T= 310 K, P = 1 atm) with OPLS-AA FF force field, using the Nosé-Hoover barostat and thermostat to control pressure and temperature, respectively. Each polymer chain was solvated with 8000 TIP3P water molecules, using 1.4 nm cut-offs for both nonbonded and electrostatic potentials, and PPPM for long-range electrostatic interactions. The simulations included a 50 ns equilibration and a 100 ns data collection run. The CG representations were generated from AA structures using the CG builder tool https://jbarnoud.github.io/cgbuilder/. For PEO, bonds, angles, and dihedrals were parametrized, while only bonds and angles were used for PBD due to the lack of a well-defined dihedral distribution for this monomer. PEO models have been previously developed for the Martini force field, and initial parameters for this molecule were adopted from literature sources[1,11,6] and refined based on our simulation results. To match the AA bonded parameters with the CG representation, a combination of optimization algorithms and trial and error were

employed until the CG distributions were in close agreement with those of the AA simulations.

The harmonic potential was used to model the bonds following the Martini force-field formulation. Bond distributions of the simulated polymers are shown in Figure S3, indicating good agreement between the CG and AA simulations. However, the bonds between TC2r-TC2r and TC2r-SN3a beads showed a bimodal behavior in the AA representation which was not captured by the CG model. As a result, the two configurations were combined into one due to the simplified phase-space sampling typical of mesoscale models. The use of non-charged Martini 3 beads in modeling can lead to underestimation of structural properties when using bond distances directly from atomistic simulations, as their attractive interactions are slightly underestimated[12]. To address this issue, bond lengths can be adjusted for a more accurate representation. One proposed solution is to slightly increase (≈ 15 %) the atomically adjusted bond length of main chains[12]. Following this suggestion, we applied this correction for SN3a-SN3a bonds and TC2r-TC2r bonds.

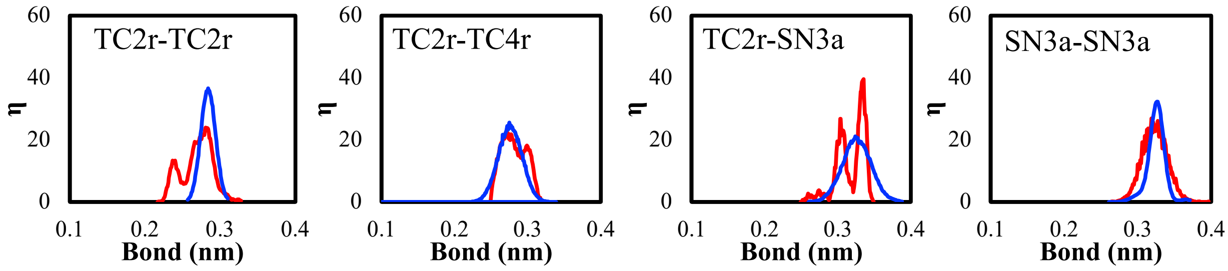

Fig. S3. AA (blue) and CG (red) bond length distributions.

The angles were also modeled using a harmonic potential, and the results are depicted in Figure S4, which shows close agreement with the atomistic reference data. Five different types of angle were considered, including main chain angles (TC2r-TC2r-TC2r, TC2r-TC2r-SN3a, TC2r-SN3a-SN3a, SN3a-SN3a-SN3a) and the main and side chain of a PBD bead with its neighboring particle (TC4r_i-TC2r_i-TC2r_{i+1}). Again, angular distribution that exhibited a bimodal behavior were combined into a single distribution in the CG scale. Simulations became unstable when the SN3a-SN3a-SN3a angles approach 180° if only an harmonic potential is used, as also reported elsewhere ([6,7]). To correct this, the constrained bending potential developed by Bulacu *et al.*[13] was applied along with the harmonic potential for this angle. With this correction, a timestep of 2 femtoseconds could be used, whereas without it, only a step of 1 femtosecond could be applied.

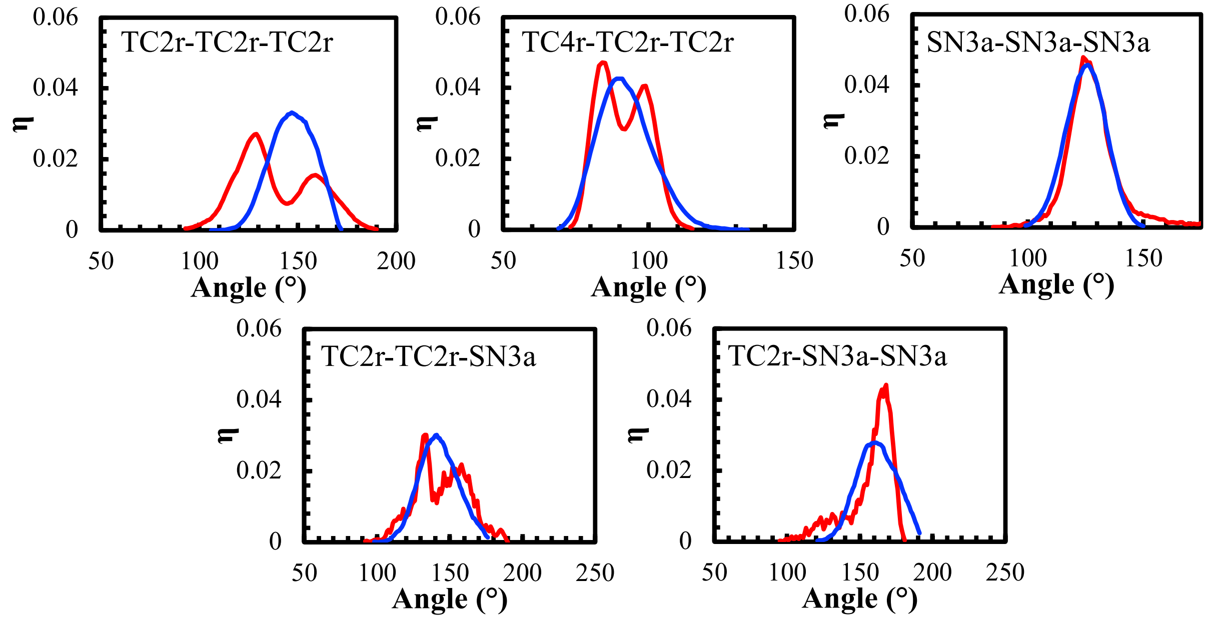

Fig. S4. AA (blue) and CG (red) angle distribution.

The dihedral distribution of PBD did not exhibit a well-defined behavior, and therefore dihedral terms were not

included for these beads. Regarding PEO, there are some peculiarities in modeling this term, such as bimodalities, and for simplicity we used the CG dihedral model implemented in the Poliply Python suite[11]. A summary of all the bond parameters is provided in Table S1.

Table S1. Bonded parameters for PBD-*b*-PEO copolymer.

Bonds parameters ($E_B = k_b(r - r_0)^2$)			
Bond type	r_0 (nm)	k_b (kJ mol^{-1} nm^{-2})	
TC2r - TC2r	0.332	50,000	
TC2r - TC4r	0.272	10,000	
TC2r - SN3a	0.330	7,000	
SN3a - SN3a	0.370	7,000	
**Angles parameters ($E_\theta = k_\theta(\theta - \theta_0)^2$)**			
Angle type	θ_0 (deg)	k_a (kJ mol^{-1} rad^{-2})	
TC2r - TC2r - TC2r	170	150	
TC4r - TC2r - TC2r	90	50	
TC2r - TC2r - SN3a	146	100	
TC2r - SN3a - SN3a	165	75	
SN3a - SN3a - SN3a	135	50	
(*constrained bending potential*)			
**Dihedral parameters ($E_\phi = k_m(1 + \cos(m\phi + \phi_0))$)**			
Dihedral type	ϕ_0 (deg)	k_m (kJ mol^{-1})	m
SN3a - SN3a - SN3a - SN3a	180	1.96	1
	0	0.18	2
	0	0.33	3
	0	0.12	4

C. Validation of the proposed model

To validate the efficacy of the proposed parameterization, we conducted calculations of the radius of gyration (R_g) and surface area of available solvent (SASA) for both the all-atom and CG representations. The SASA was calculated based on the system used for parameterization, as it represents the polymers fully immersed in water. Meanwhile, the R_g was calculated through independent simulations of 30 polymer chains in a simulation box to assess the ability of the proposed model to capture the degree of packing of the bulk polymer. It is important to note that both bonded and non-bonded parameters can impact the R_g and SASA, and the presented results are based on the final set of parameters utilized in the simulations.

The radius of gyration is a measure of the overall size of the molecule and is defined by Eq. S1:

$$R_g = \sqrt{\frac{1}{N}\sum_{i=1}^{N} |r(i) - r_{com}|^2} \tag{S1}$$

where $r(i)$ and r_{com} are the coordinates of atom i and the center of mass of the molecule, respectively, and N is the number of atoms. Figure S5 illustrates the radius of gyration of the three polymer chains systems studied during parameterization. The excellent agreement between the distributions of the AA and CG trajectories suggests that the Martini parameterization proposed in this work accurately represents the overall size of the molecule.

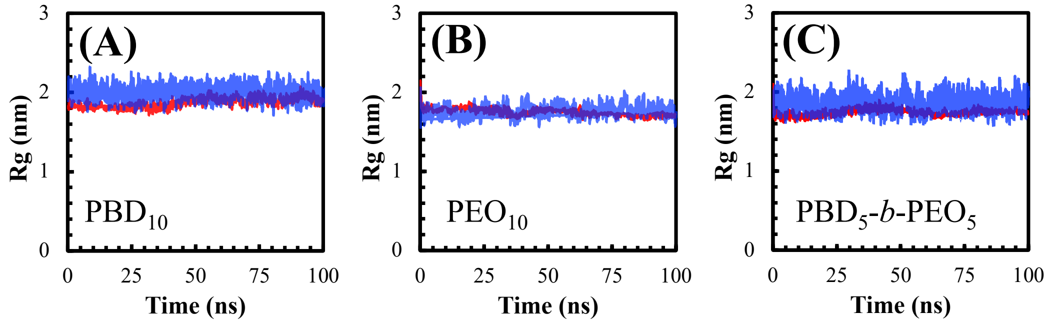

Fig. S5. Radius of gyration of AA (blue) and CG (red) representations of (A) PBD, (B) PEO, and (C) PBD-PEO.

SASA measures the area of a molecule available to establish contact with its surrounding solvent or media. As polar and nonpolar residues are being parameterized, SASA is a valuable metric for evaluating their behavior in a solvated environment. According to Lee and co-workers[14], SASA is calculated based on the atomic van der Waals radius by:

$$SASA = \sum_{i=1}^{N} \frac{R}{\sqrt{(R^2 - Z_i^2)}} L_i \left(\frac{\Delta Z}{2} + \Delta' Z \right) \tag{S2}$$

where R is the radius, L_i is the length of the arc drawn in a given section i, Z_i is the perpendicular distance of the section i to the center of the sphere, ΔZ is the space between sections and $\Delta' Z$ is $(\Delta Z/2)$ or $(R - Z_j)$ (whichever is smaller). The SASA distributions of the three polymers were evaluated for both their AA and CG representations (Fig. S6). In general, the CG model underestimated the SASA values by about 5 %, which is in good agreement with the trends reported by the Martini developers[3]. As this is a small deviation, our analysis of the radius of gyration (R_g) and SASA provides strong evidence that the CG model is a good representation of the AA structure.

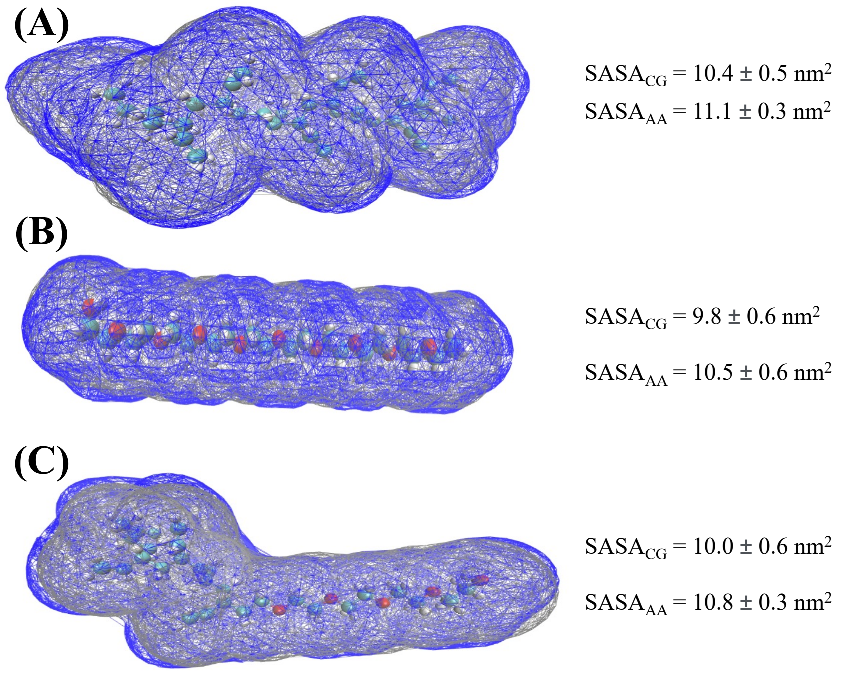

Fig. S6. Connolly surfaces of (A) PBD, (B) PEO, and (C) PBD-*b*-PEO, where the blue and gray contours represent the estimated areas for the AA and CG models.

2. INCORPORATION OF PEPTIDE

A. Umbrella Sampling

Umbrella sampling (US) simulations were conducted to analyze the interactions of HVs with a peptide model as discussed in the main text. Figure S7 shows the system representation with the peptide embedded in the center of the membrane and histograms of the US windows. The good overlap between adjacent windows suggests that the simulations were properly spaced and converged.

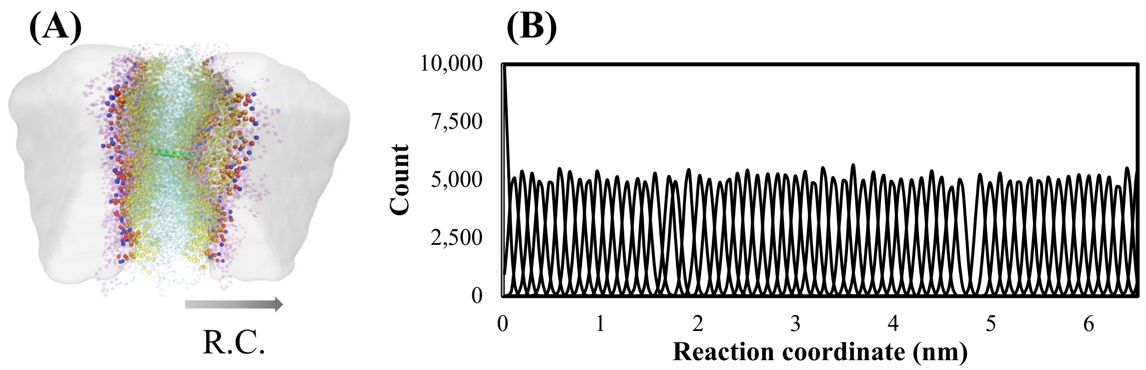

Fig. S7. (A) Representation of a system used in the US simulations and (B) Histogram showing the overlap between adjacent windows.

The peptide can be pulled out of the membrane from both its carboxyl and amine termini. While the main manuscript reported results for the *N* terminus, Figure S8 illustrates the results for both termini. The curves obtained for each terminus differ slightly, primarily due to differences in the peptide's behavior within the membrane. The data indicate that the energy in the center of the bilayer and in the bulk water are equal for both termini, within the error margin. The findings suggest that the charged termini primarily influence the behavior of the peptide within the bilayer. These results are in line with the findings of Yue *et al.*[15], who proposed that the *N* terminus is thermodynamically preferred for peptide reconstitution. In line with these results, our research revealed an additional energy barrier for the *C* terminus.

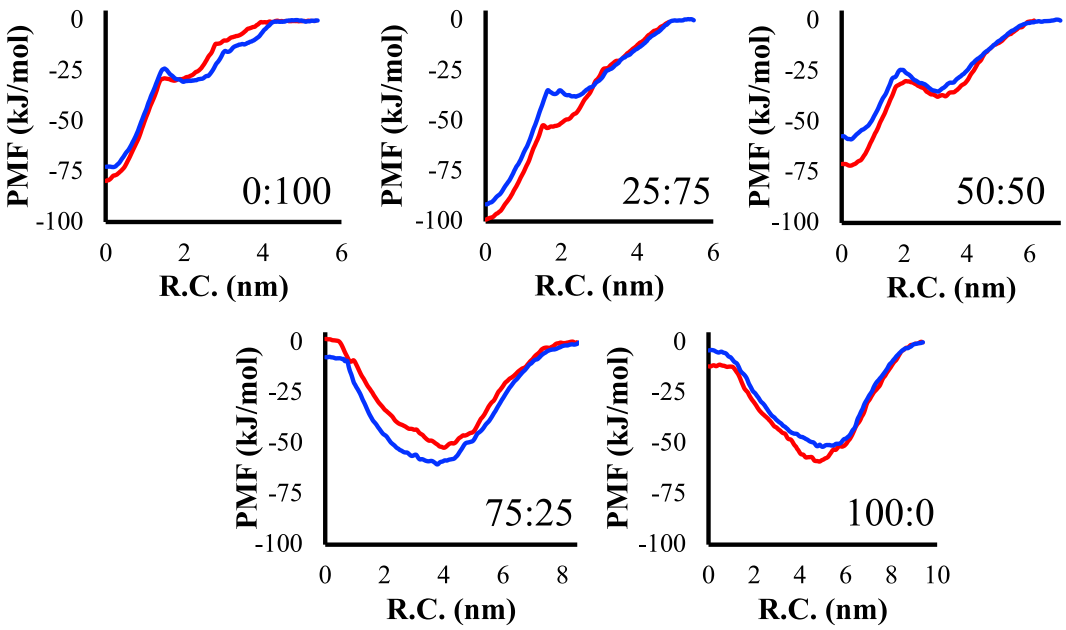

Fig. S8. Potential of mean force for different vesicles (lipid:polymer %) for the peptide being pulled from the *N* (red) and *C* (blue) terminus.

B. Equilibrium simulations

We further investigated the conformations of the transmembrane peptide that showed a favorable transmembrane configuration using equilibrium simulations. To examine local properties, we discretized the membrane into a grid with a resolution of 0.5 nm along the x and y axes. We applied the nonlocal weighted averaging method proposed by[16] to ensure that no empty cells were present. This approach involves determining the local value of a variable by averaging the values of that property for all molecules within a radius of σ from the center of the cell, known as the horizon. The local value of a generic variable $f(x_i)$ is then determined by the following relation:

$$f(\bar{x}_i) = \frac{1}{\sum_{j=1}^{N_c} w(r_{x_j})} \left[\sum_{j=1}^{N_c} w(r_{x_j}) f(x_j) \right] \tag{S3}$$

where $w(r_{x_j})$ is the weight assigned to a particle at radius r_{x_j} away from the center of the element $\bar{x}_i$, $f(x_j)$ is the value of the measured property for this particle and N_c is the number of particles that lie within the horizon. The weights assigned to each molecule are given by S4:

$$w(r_{x_j}) = \frac{1}{1 - e^{-\alpha}} \left[e^{-\alpha r_{x_j}/\delta} - e^{-\alpha} \right] \tag{S4}$$

It can be observed that the average weight of molecules decreases exponentially with distance from the center at a rate defined by α. The value of α can be adjusted to achieve a desired level of resolution and, in this study, $\alpha = 8$ and $\sigma = 0.5$ nm, which have been found to provide an adequate sampling by preventing empty cells. The profiles for the total, POPC, and polymer-normalized density, membrane thickness, and POPC order parameter obtained at all analyzed temperatures and bilayer compositions are shown in Figures S9-S13.

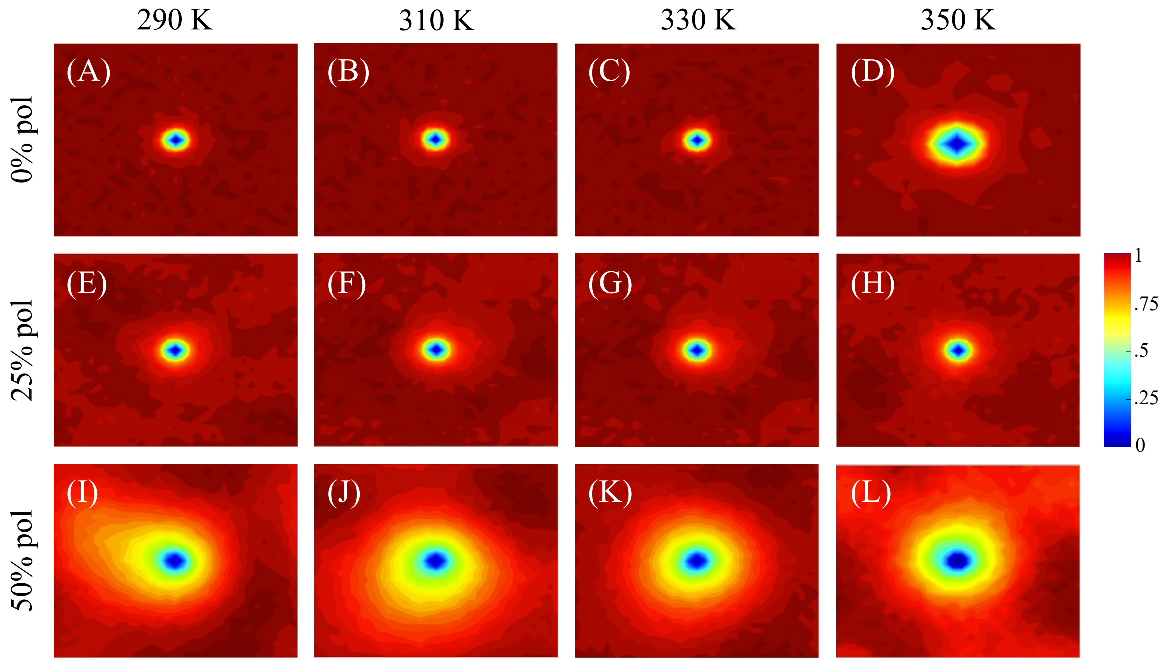

Fig. S9. Membrane normalized density profiles as a function of temperature (columns) and polymer concentration (rows): (A) 290 K - 0(polymer):100(lipid), (B) 310 K - 0:100, (C) 330 K - 0:100, (D) 350 K - 0:100, (E) 290 K - 25:75, (F) 310 K - 25:75, (G) 330 K - 25:75, (H) 350 K - 25:75, (I) 290 K - 50:50, (J) 310 K - 50:50, (K) 330 K - 50:50, (L) 350 K - 50:50.

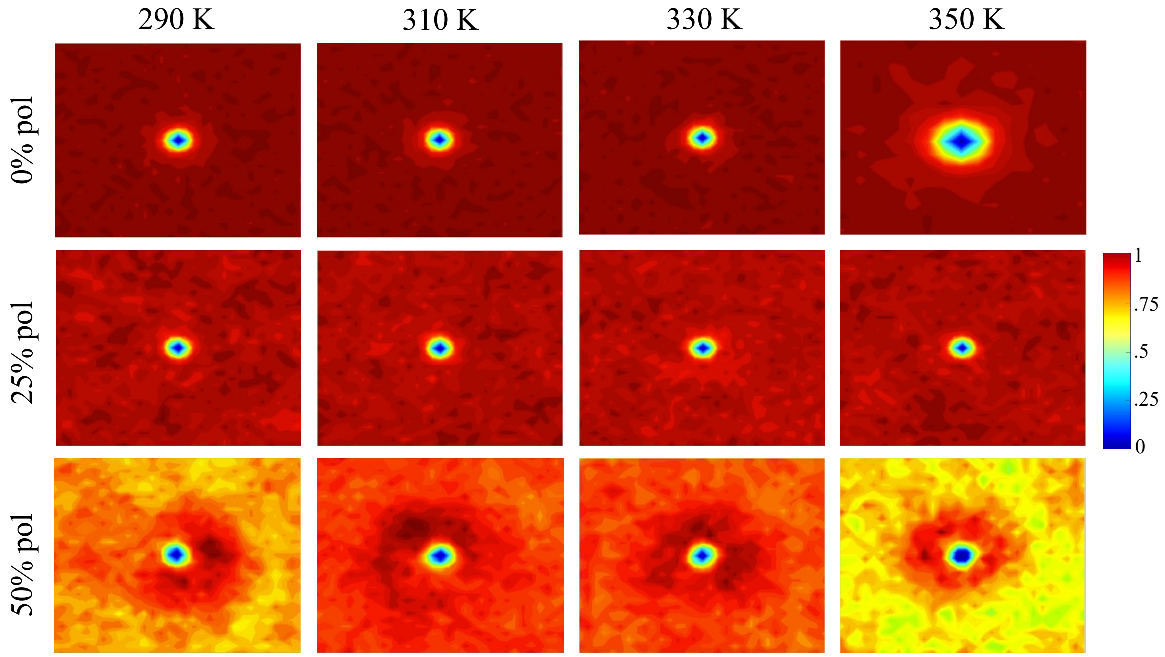

Fig. S10. POPC normalized density profiles as function of temperature (columns) and polymer concentration (rows): (A) 290 K - 0(polymer):100(lipid), (B) 310 K - 0:100, (C) 330 K - 0:100, (D) 350 K - 0:100, (E) 290 K - 25:75, (F) 310 K - 25:75, (G) 330 K - 25:75, (H) 350 K - 25:75, (I) 290 K - 50:50, (J) 310 K - 50:50, (K) 330 K - 50:50, (L) 350 K - 50:50.

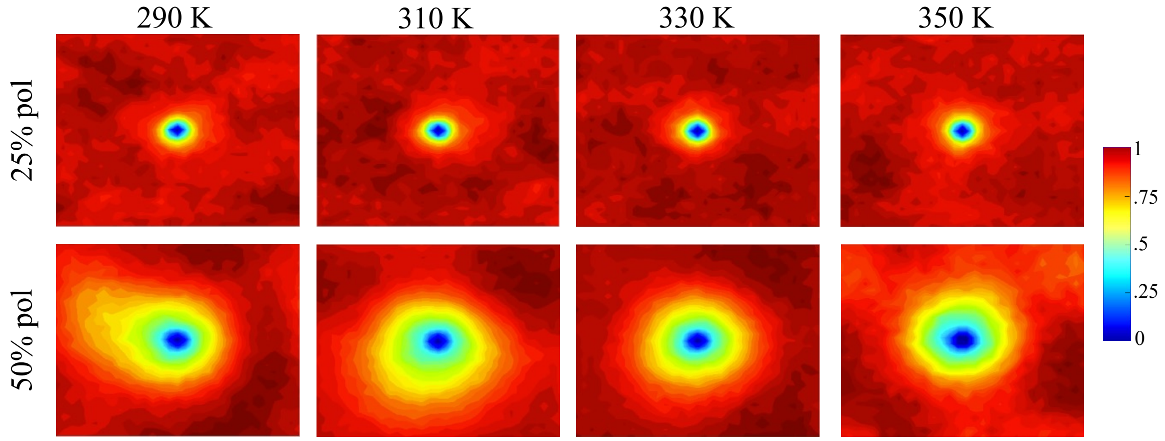

Fig. S11. PBD_{22}-*b*-PEO_{14} normalized density profiles as function of temperature (columns) and polymer concentration (rows): (A) 290 K - 0(polymer):100(lipid), (B) 310 K - 0:100, (C) 330 K - 0:100, (D) 350 K - 0:100, (E) 290 K - 25:75, (F) 310 K - 25:75, (G) 330 K - 25:75, (H) 350 K - 25:75, (I) 290 K - 50:50, (J) 310 K - 50:50, (K) 330 K - 50:50, (L) 350 K - 50:50.

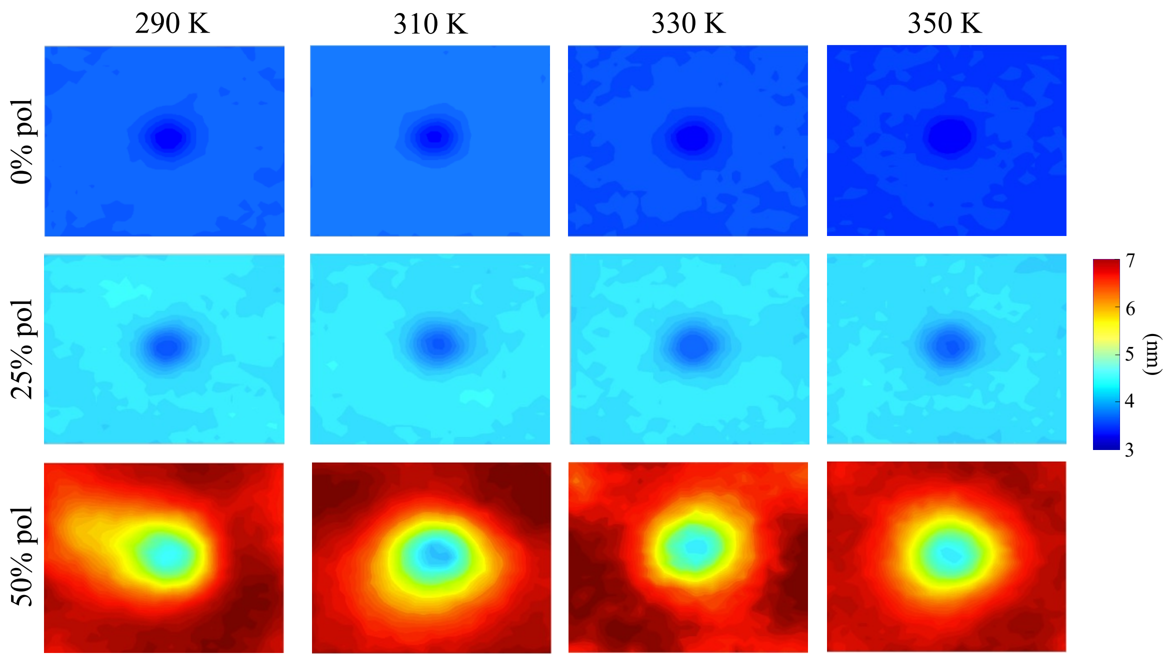

Fig. S12. Membrane thickness as a function of temperature (columns) and polymer concentration (rows): (A) 290 K - 0(polymer):100(lipid), (B) 310 K - 0:100, (C) 330 K - 0:100, (D) 350 K - 0:100, (E) 290 K - 25:75, (F) 310 K - 25:75, (G) 330 K - 25:75, (H) 350 K - 25:75, (I) 290 K - 50:50, (J) 310 K - 50:50, (K) 330 K - 50:50, (L) 350 K - 50:50.

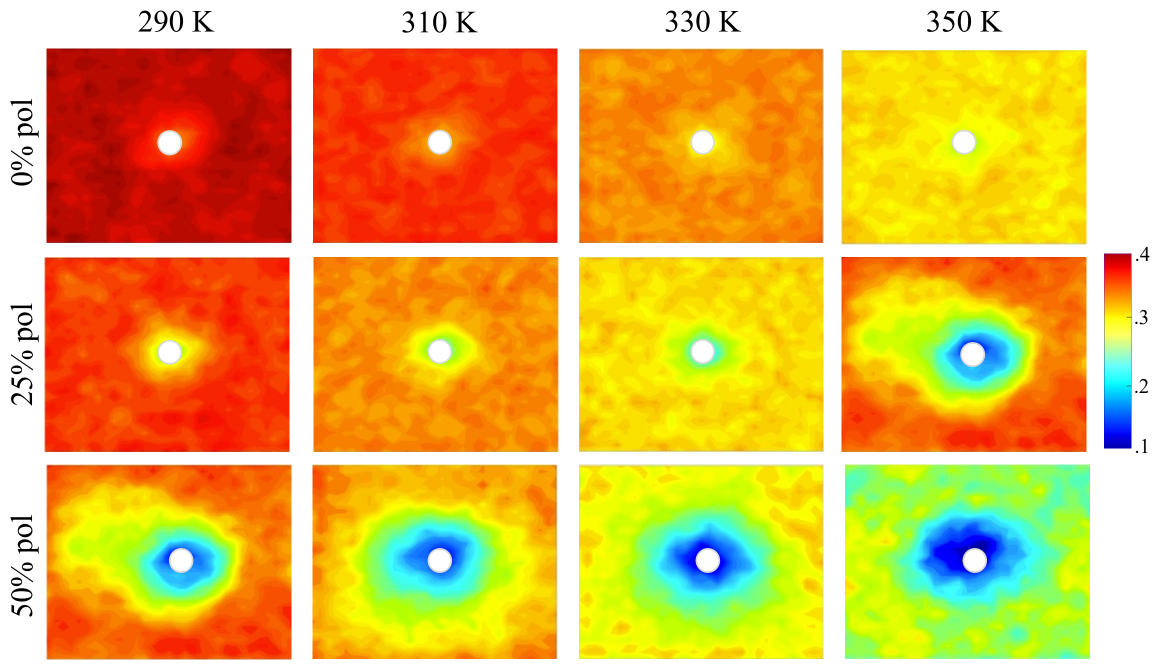

Fig. S13. POPC order parameter as a function of temperature (columns) and polymer concentration (rows): (A) 290 K - 0(polymer):100(lipid), (B) 310 K - 0:100, (C) 330 K - 0:100, (D) 350 K - 0:100, (E) 290 K - 25:75, (F) 310 K - 25:75, (G) 330 K - 25:75, (H) 350 K - 25:75, (I) 290 K - 50:50, (J) 310 K - 50:50, (K) 330 K - 50:50, (L) 350 K - 50:50.

3. DYNAMIC LIGHT SCATTERING MEASUREMENTS

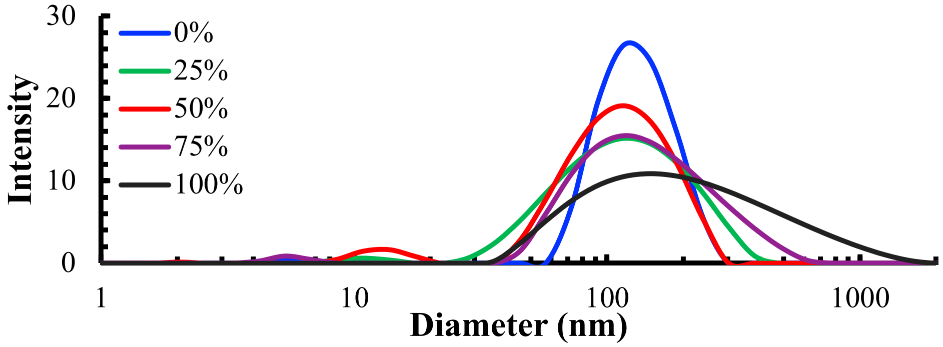

Fig. S14. Vesicle diameter distribution for various polymer concentrations

4. FIRST MOMENT OF THE PRESSURE PROFILE

Table S2. First moment of pressure profiles for all temperatures and membrane compositions.

	0:100	25:75	50:50	75:25	100:0
290	-0.016	-0.055	-0.027	0.057	0.025
310	-0.015	-0.133	0.035	0.201	0.483
330	-0.010	0.280	0.116	-0.148	-0.293
350	-0.039	0.028	0.002	-0.293	0.184

5. DIFFUSIVITY FOR POPC AND PBD_{22}-*B*-PEO_{14}

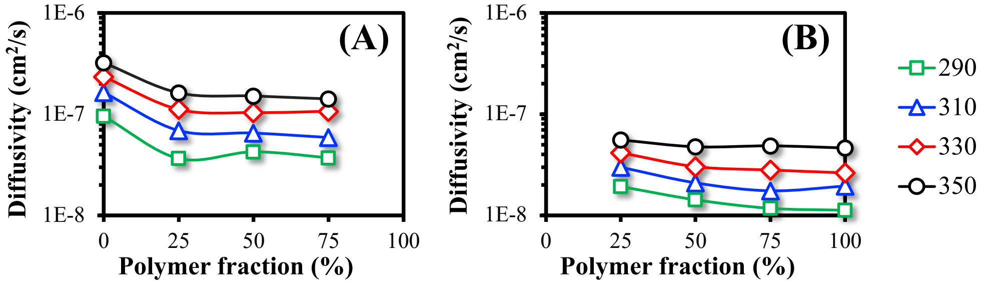

Fig. S15. Lateral diffusion coefficient at varied temperatures for (A) POPC and (B) PBD_{22}-*b*-PEO_{14}.

6. COMPLETE ELECTRON DENSITY PROFILES FOR ALL COMPONENTS OF THE SYSTEMS

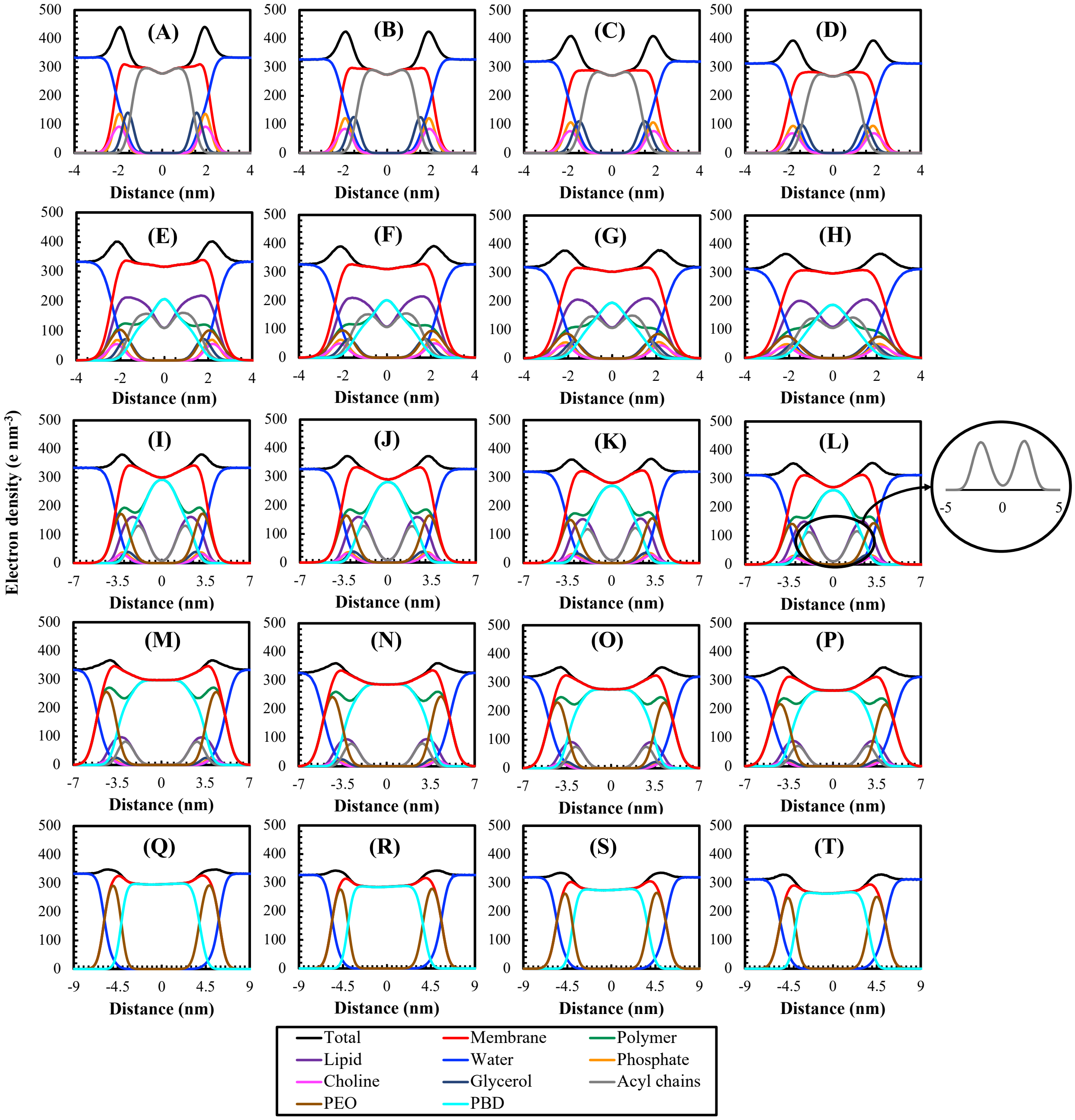

Fig. S16. Electron density profiles for membranes at different temperatures (columns) with varying polymer concentrations (rows): (A) 290 K - 0(polymer):100(lipid), (B) 310 K - 0:100, (C) 330 K - 0:100, (D) 350 K - 0:100, (E) 290 K - 25:75, (F) 310 K - 25:75, (G) 330 K - 25:75, (H) 350 K - 25:75, (I) 290 K - 50:50, (J) 310 K - 25:75, (K) 330 K - 50:50, (L) 350 K - 50:50, (M) 290 K - 75:25, (N) 310 K - 75:25, (O) 330 K - 75:25, (P) 350 K - 75:25, (Q) 290 K - 100:0, (R) 310 K - 100:0, (S) 330 K - 100:0, (T) 350 K - 100:0. The detail on the right presents a separate profile for the acyl chains in a 50:50 membrane, revealing the presence of acyl chains both at the center and at the extremities of the bilayer's hydrophobic core.

7. ADDITIONAL ANALYSIS REGARDING THE STRUCTURE OF THE MEMBRANE

To analyze the degree of membrane interdigitation, we utilized the MembPlugin developed by Ref.[17]. This tool employs a correlation-based approach to evaluate the overlap of the mass distribution of the two leaflets along the membrane normal.

The fraction of overlap, denoted by the symbol I_ρ, is computed using the equation $I_\rho^2 = 4\int \rho_a(z)\rho_b(z)dz / \int[\rho_a(z) + \rho_b(z)]^2 dz$, where ρ_a and ρ_b represent the density profiles of the two leaflets. The resulting interdigitation values at a temperature of 310 K are presented in Figure S17 for the complete membrane, as well as for the POPC and PBD_{22}-*b*-PEO_{14} molecules. At low polymer concentrations, the degree of interdigitation increases due to an increase in the fraction of polymers that interdigitate. Simultaneously, the lateral area of the membrane expands, which has been discussed in detail in the main manuscript. The combined effect of these phenomena is responsible for the observed thin membrane configuration, which is further elaborated on in the main document. It is noteworthy that both the 25:75 and 50:50 cases exhibit an increase in membrane interdigitation of approximately 25% compared to membranes with higher polymer concentration. Interestingly, the average lateral area of the membrane also increases by a similar amount in these compositions. These findings suggest that interdigitation and collapsed structures may have comparable influence on the 'thin' configuration of the membrane Furthermore, the results indicate that as the polymer fraction increases, the interdigitation of POPC decreases, indicating that more lipids are concentrated in the hydrophilic interface of the thicker conformation.

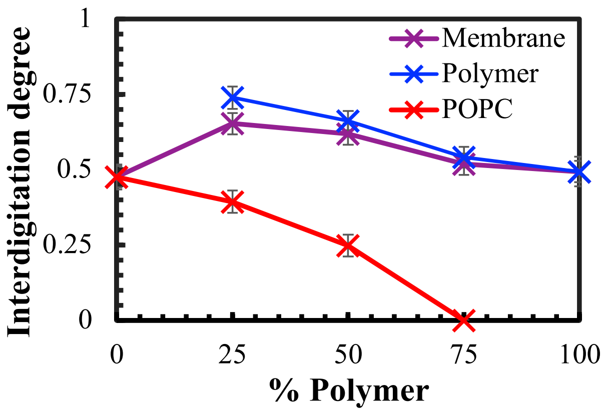

Fig. S17. Fraction of overlap between leaflets for different HVs at 330 K.

Figure S18 displays the phosphate-phosphate distance of a 50:50 HV. Thin regions (with a thickness of approximately 40 Å) are present throughout the membrane, with greater concentration observed in the upper part. It is noteworthy that this configuration constitutes only about 20% of the total area and is embedded in a much thicker membrane (approximately 70 Å), providing further evidence of the existence of two distinct configurations in this condition.

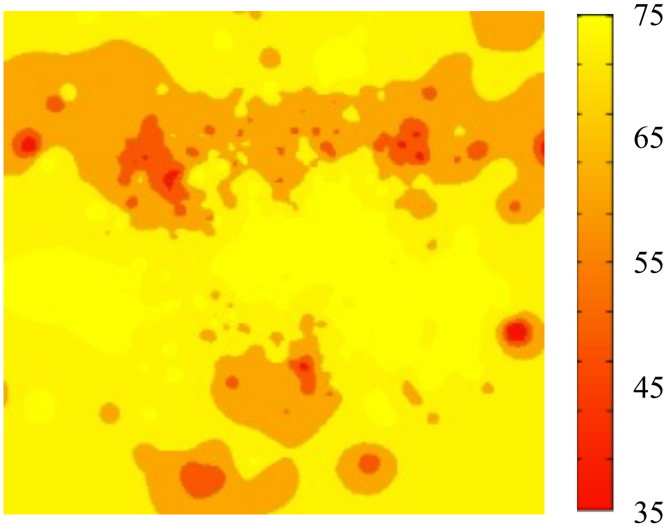

Fig. S18. Membrane thickness profile for a 50:50 HV at 330 K showing the 'thin' and 'thick' regions as discussed in the main manuscript.

8. PARAMETERS OF THE 4PL MODEL OBTAINED FROM REGRESSION ANALYSIS OF THE FLUORESCENCE EXPERIMENTS

Table S3. 4PL parameters and regression coefficient for varied polymer/lipid ratios.

	b	c	R^2
0:100	0.0058	5.89	0.979
25:75	0.0045	4.10	0.990
50:50	0.0069	1.29	0.991
75:25	0.0073	1.06	0.962
100:0	0.0667	4.11	0.978

REFERENCES

[1] D. A. Grillo, J. M. R. Albano, E. E. Mocskos, J. C. Facelli, M. Pickholz, and M. B. Ferraro, "Diblock copolymer bilayers as model for polymersomes: A coarse grain approach," The J. Chem. Phys. **146**, 244904 (2017).

[2] S. J. Marrink, H. J. Risselada, S. Yefimov, D. P. Tieleman, and A. H. de Vries, "The MARTINI force field: coarse grained model for biomolecular simulations," The J. Phys. Chem. B **111**, 7812–7824 (2007).

[3] P. C. T. Souza, R. Alessandri, J. Barnoud, S. Thallmair, I. Faustino, F. Grünewald, I. Patmanidis, H. Abdizadeh, B. M. H. Bruininks, T. A. Wassenaar, P. C. Kroon, J. Melcr, V. Nieto, V. Corradi, H. M. Khan, J. Domański, M. Javanainen, H. Martinez-Seara, N. Reuter, R. B. Best, I. Vattulainen, L. Monticelli, X. Periole, D. P. Tieleman, A. H. de Vries, and S. J. Marrink, "Martini 3: a general purpose force field for coarse-grained molecular dynamics," Nat. Methods **18**, 382–388 (2021).

[4] H. Lee, A. H. de Vries, S.-J. Marrink, and R. W. Pastor, "A coarse-grained model for polyethylene oxide and polyethylene glycol: Conformation and hydrodynamics," The J. Phys. Chem. B **113**, 13186–13194 (2009).

[5] G. Rossi, L. Monticelli, S. R. Puisto, I. Vattulainen, and T. Ala-Nissila, "Coarse-graining polymers with the MARTINI force-field: polystyrene as a benchmark case," Soft Matter **7**, 698–708 (2011).

[6] G. Rossi, P. F. J. Fuchs, J. Barnoud, and L. Monticelli, "A coarse-grained MARTINI model of polyethylene glycol and of polyoxyethylene alkyl ether surfactants," The J. Phys. Chem. B **116**, 14353–14362 (2012).

[7] F. Grunewald, G. Rossi, A. H. de Vries, S. J. Marrink, and L. Monticelli, "Transferable MARTINI model of poly(ethylene oxide)," The J. Phys. Chem. B **122**, 7436–7449 (2018).

[8] R. Seneviratne, R. Catania, M. Rappolt, L. J. C. Jeuken, and P. A. Beales, "Membrane mixing and dynamics in hybrid POPC/poly(1, 2-butadiene-block-ethylene oxide) (PBd-b-PEO) lipid/block co-polymer giant vesicles," Soft Matter **18**, 1294–1301 (2022).

[9] R. Seneviratne, G. Coates, Z. Xu, C. Cornell, R. Thompson, A. Sadeghpour, D. Maskell, L. Jeuken, M. Rappolt, and P. Beales, "High resolution membrane structures within hybrid lipid-polymer vesicles revealed by combining x-ray scattering and electron microscopy," a (2022).

[10] G. Srinivas, D. E. Discher, and M. L. Klein, "Self-assembly and properties of diblock copolymers by coarse-grain molecular dynamics," Nat. Mater. **3**, 638–644 (2004).

[11] F. Grünewald, R. Alessandri, P. C. Kroon, L. Monticelli, P. C. Souza, and S. J. Marrink, "Polyply; a python suite for facilitating simulations of (bio-) macromolecules and nanomaterials," Nat. Commun. **13**, 68 (2022).

[12] F. Grunewald, G. Rossi, A. H. de Vries, S. J. Marrink, and L. Monticelli, "Transferable MARTINI model of poly(ethylene oxide)," The J. Phys. Chem. B **122**, 7436–7449 (2018).

[13] M. Bulacu, N. Goga, W. Zhao, G. Rossi, L. Monticelli, X. Periole, D. P. Tieleman, and S. J. Marrink, "Improved angle potentials for coarse-grained molecular dynamics simulations," J. Chem. Theory Comput. **9**, 3282–3292 (2013).

[14] B. Lee and F. Richards, "The interpretation of protein structures: Estimation of static accessibility," J. Mol. Biol. **55**, 379–IN4 (1971).

[15] T. Yue, M. Sun, S. Zhang, H. Ren, B. Ge, and F. Huang, "How transmembrane peptides insert and orientate in biomembranes: a combined experimental and simulation study," Phys. Chem. Chem. Phys. **18**, 17483–17494 (2016).

[16] A. K. Chaurasia, A. M. Rukangu, M. K. Philen, G. D. Seidel, and E. C. Freeman, "Evaluation of bending modulus of lipid bilayers using undulation and orientation analysis," Phys. Rev. E **97** (2018).

[17] R. Guixà-González, I. Rodriguez-Espigares, J. M. Ramírez-Anguita, P. Carrió-Gaspar, H. Martinez-Seara, T. Giorgino, and J. Selent, "MEMBPLUGIN: studying membrane complexity in VMD," Bioinformatics **30**, 1478–1480 (2014).
